# Nitrogen Fertilizer Formulations Modulate the Yield–Cadmium Trade-Off in Rice Through Rhizosphere Processes and Translocation Nodes

**DOI:** 10.3390/plants15172618

**Published:** 2026-08-27

**Authors:** Yusheng Zhang, Hejun Ao, Xilin Fang, Xing Li, Hongyu Zhang, Ting Zhong, Xuefei Tian, Xianglan Zeng, Wupeng Ji, Min Luo

**Affiliations:** 1 Modern Agricultural and Forestry Engineering College, Ganzhou Green Leafy Vegetables Technology Innovation Center*,* Ganzhou Polytechnic, Ganzhou 341008, China; zhangyusheng@gzpt.edu.cn (Y.Z.);; 2 College of Agronomy, Hunan Agricultural University, Changsha 410128, China; 3 National Engineering and Technology Research Center for Red Soil Improvement, Jiangxi Institute of Red Soil and Germplasm Resources, Key Laboratory of Arable Land Improvement and Quality Improvement of Jiangxi Province, Nanchang 331717, China

**Keywords:** rice (*Oryza sativa* L.), Cd, nitrogen form, ammonium nitrogen, translocation

## Abstract

Cadmium (Cd) contamination in paddy soils represents a major risk to global food security and human health because Cd can readily enter the food chain through rice consumption. Therefore, clarification of the key processes and mechanisms by which agronomic practices regulate Cd accumulation in rice is essential. Based on integrated two-year pot and field experiments, we showed that different nitrogen fertilizer formulations, including nitrate-N (N), ammonium-N (A), and urea (U), had distinct effects on Cd accumulation and grain yield in rice. The N treatment reduced Cd concentrations in brown rice by 25.29% to 70% in the pot experiment and by 4.25% to 89.97% in the field experiment but decreased grain yield by 5% to 25%. By contrast, the A treatment increased Cd concentrations in brown rice by 17.86% to 58.62%, while maintaining or slightly increasing grain yield (−3% to +5%), and the U treatment showed intermediate responses. These responses were mainly associated with nitrogen-induced shifts in rhizosphere chemistry, especially changes in soil Cd availability linked to pH and exchangeable H^+^, although unmeasured redox-related processes may have also contributed under flooded conditions. Further analyses of internal Cd distribution and translocation, together with exploratory random forest modeling, suggested that Cd transport efficiency at key stem internodes and Cd redistribution from the panicle to the grain were important regulatory nodes associated with Cd concentrations in brown rice. These regulatory nodes were markedly affected by fertilizer formulation. Overall, our results describe a continuous pathway from rhizosphere Cd availability to internal transport and partitioning, through which nitrogen fertilizer formulations regulate Cd accumulation in rice. This study aimed to clarify how different nitrogen fertilizer formulations regulate the trade-off between grain yield and Cd accumulation in rice, based on the hypothesis that these formulations differentially modify rhizosphere chemistry and Cd bioavailability, that specific stem nodes contribute to control of grain Cd accumulation, and that the main regulatory processes differ between pot and field systems. This study provides s a scientific basis for developing practical nitrogen-management strategies to support safe rice production in Cd-contaminated paddy fields.

## 1. Introduction

Cd is a non-essential heavy metal (HM) with high mobility and strong biological toxicity, and it is widely present in soils. Rapid industrialization has increased Cd inputs into agricultural soils through mining, smelting, traffic emissions, and the use of Cd-containing fertilizers and sewage sludge. Consequently, Cd contamination in farmland has shifted from a localized concern to a more widespread environmental issue [1,2]. Cd can migrate through the soil–plant system and gradually accumulate along the food chain, while also disturbing soil microbial communities and nutrient cycling, thereby creating long-term risks for agroecosystem stability [3]. Because of its high mobility, Cd is easily solubilized and absorbed by plants. Once Cd enters the plow layer, it persists for long periods and is difficult to remove using conventional agronomic practices, creating a serious and lasting threat to sustainable agriculture and human health [4].

Rice (*Oryza sativa* L.), a staple food for nearly half of the world population, has a central role in global food security. Because of flooded cultivation, high root adsorptive capacity, and strong sensitivity to Cd, rice is a major pathway for human dietary Cd intake [5]. In contaminated or slightly polluted regions, rice grains remain the main dietary source of Cd. Previous studies have reported that rice may contribute 40–70% or more of total dietary Cd intake among populations across East and Southeast Asia [6,7]. Long-term exposure to Cd is associated with kidney dysfunction, bone disorders, and cardiovascular diseases, and Cd has been classified as a Group I human carcinogen by the International Agency for Research on Cancer (IARC) [8]. Therefore, reducing Cd accumulation in rice, especially in brown rice, has become an important priority in agricultural and environmental health research. Several key transporters involved in these processes have gradually been identified. For example, *OsNRAMP5* and *OsIRT1* are mainly responsible for Cd uptake by roots [9,10], *OsHMA2* mediates xylem loading and root-to-shoot translocation [11], and *OsLCT1* is involved in phloem-mediated Cd transport to grains [12,13]. These molecular components provide important mechanistic reference points for understanding how agronomic practices, including nitrogen fertilization, may regulate Cd accumulation in rice.

The processes of Cd uptake, root retention, shoot translocation, and final allocation to grains in rice are controlled by complex interactions among rhizosphere conditions, soil physicochemical properties, and plant physiology [11,14,15]. Among agronomic practices, fertilization management is highly controllable and strongly influences Cd speciation, rhizosphere chemistry, and plant uptake processes [16]. In particular, nitrogen fertilization can change Cd behavior by modifying rhizosphere pH, iron plaque formation, root activity, and the expression of metal transporter genes [9,17,18,19,20]. Importantly, rice responses to Cd, including uptake and internal transport, differ notably among nitrogen fertilizer formulations [21]. In flooded paddy soils, Cd mobility is also closely linked to redox conditions, Fe/Mn cycling, dissolved organic carbon, and the formation or dissolution of iron plaque on root surfaces [7,22]. Thus, the effects of nitrogen fertilizer formulation on Cd accumulation may involve pH-mediated chemical processes as well as redox-sensitive biogeochemical reactions.

However, a unified understanding of the complete pathway linking nitrogen fertilizer formulation, rhizosphere dynamics, internal Cd translocation, and final grain accumulation is still limited, particularly in studies that combine controlled pot experiments with field validation. To address this gap, we conducted a comprehensive two-year study that combined pot and field experiments using Cd-contaminated paddy soils. This study aimed to determine how different nitrogen fertilizer formulations regulate Cd uptake, translocation, and accumulation in rice grains. We hypothesized that nitrogen fertilizer formulations distinctly modify the rhizosphere chemical environment and thereby influence Cd bioavailability and root uptake; that Cd accumulation in brown rice is controlled more strongly by nitrogen-mediated Cd distribution and translocation at specific stem nodes than by total plant Cd uptake alone; and that the main regulatory steps controlling grain Cd accumulation differ between pot and field cultivation systems. Compared with previous studies that mainly focused on either soil Cd availability or plant Cd uptake, this study integrates rhizosphere chemistry, internal Cd translocation nodes, and pot-field validation into a pathway-based framework. Ultimately, this study provides deeper mechanistic insight into nitrogen-Cd interactions and supports practical agronomic strategies for reducing grain Cd risk while maintaining stable yields in Cd-contaminated paddy systems.

## 2. Results

### 2.1. Effects of Nitrogen Fertilizer Formulation on Rice Yield and Brown Rice Cd Concentration

**Figure 1:**
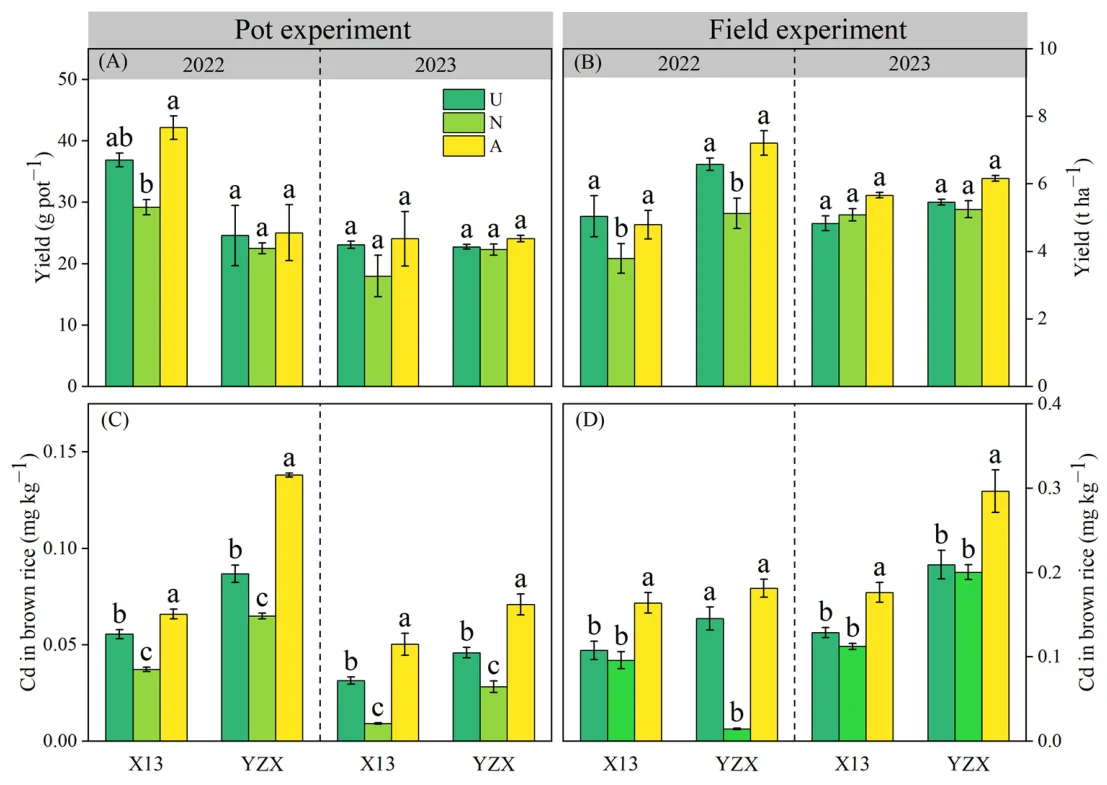
Effects of nitrogen fertilizer formulations on rice yield and Cd concentrations in brown rice (BR) under pot and field conditions. (**A**) Pot experiment yield (2022–2023); (**B**) field experiment yield (2022–2023); (**C**) BR Cd concentration in the pot experiment (2022–2023); (**D**) BR Cd concentration in the field experiment (2022–2023). U, urea; N, nitrate-N; A, ammonium-N. X13, Xiangwanxian 13 hao; YZX, Yuzhenxiang. Bars indicate the means ± standard error (SE; *n* = 3). Different lowercase letters above the bars indicate significant differences among nitrogen treatments within each cultivar and cultivation condition (Tukey’s test, *p* < 0.05).

Both pot and field experiments consistently indicated that nitrogen fertilizer formulation significantly affected grain yield and Cd concentration in brown rice (BR) (Figure 1). Compared with the urea (U) treatment, the nitrate-N (N) treatment consistently produced the lowest grain yield, but it also markedly reduced BR Cd concentration. For example, under field conditions in 2022, the N treatment significantly reduced the grain yield of the cultivars Xiangwanxian 13 hao (X13) and Yuzhenxiang (YZX) by 24.80% and 22.19%, respectively, compared with the U treatment. In contrast, the ammonium-N (A) treatment maintained or slightly increased yield, with no significant difference from the U treatment, while significantly increasing BR Cd concentration.

Specifically, under pot conditions in 2022–2023, the N treatment reduced BR Cd concentrations in X13 and YZX by 33.93% to 70% and 25.29% to 39.13%, respectively, relative to the U treatment. Conversely, the A treatment increased BR Cd concentrations by 17.86% to 38% in X13 and by 54.35% to 58.62% in YZX. In the field experiments during the same period, the N treatment decreased BR Cd concentrations in both cultivars by 4.25% to 89.97% compared with the U treatment, whereas the A treatment increased Cd concentrations by 36.96% to 54.35%.

Because Cd distribution patterns within plants differed among years, cultivars, and cultivation systems, the following analyses focused on overall Cd distribution and translocation characteristics, together with a systematic evaluation of nitrogen regulation.

### 2.2. Effects of Nitrogen Fertilizer Formulation on Rice Yield Components

**Figure 2:**
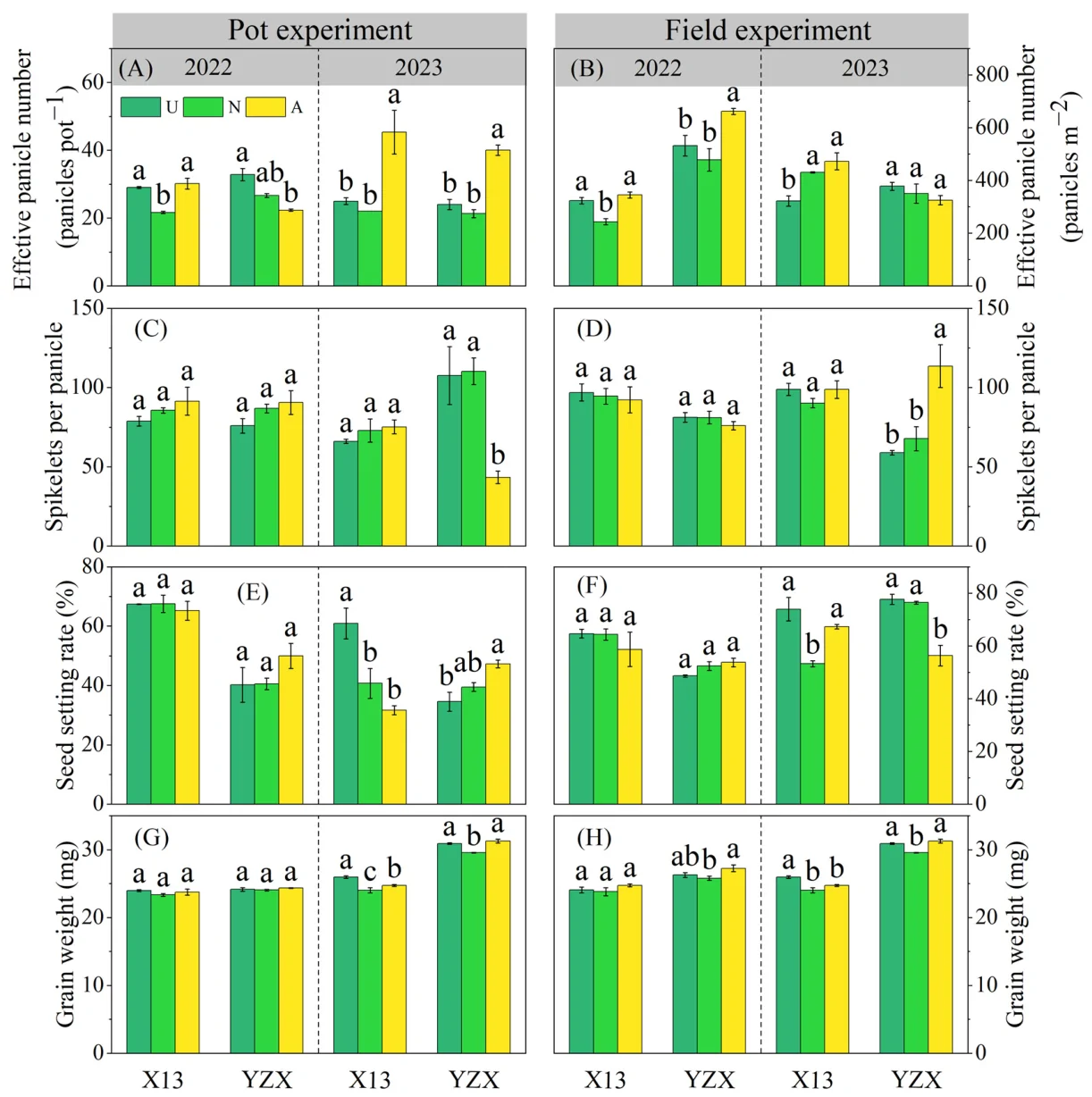
Effects of different nitrogen fertilizer formulations on rice yield components under pot and field conditions. (**A**) Effective panicle number in the pot experiment (2022–2023); (**B**) effective panicle number in the field experiment (2022–2023); (**C**) spikelets per panicle in the pot experiment (2022–2023); (**D**) spikelets per panicle in the field experiment (2022–2023); (**E**) seed setting rate in the pot experiment (2022–2023); (**F**) seed setting rate in the field experiment (2022–2023); (**G**) grain weight in the pot experiment (2022–2023); (**H**) grain weight in the field experiment (2022–2023). U, urea; N, nitrate-N; A, ammonium-N. X13, Xiangwanxian 13 hao; YZX, Yuzhenxiang. Bars indicate the means ± standard error (SE; *n* = 3). Different lowercase letters above the bars indicate significant differences among the nitrogen treatments within each cultivar and cultivation condition (Tukey’s test, *p* < 0.05).

Under pot conditions, the effects of nitrogen fertilizer formulation on yield components were mainly reflected in the effective panicle number, whereas grains per panicle, seed setting rate, and grain weight responded less clearly to the treatments (Figure 2A,C,E,G).

In 2022, the effective panicle number of Xiangwanxian 13 hao (X13) under nitrate-N (N) treatment was significantly lower than that under the urea (U) and ammonium-N (A) treatments by 33.83% and 39.22%, respectively. For Yuzhenxiang (YZX), the effective panicle number under the N treatment decreased by 23.10% compared with the U treatment, whereas it decreased by 47.02% under the A treatment relative to the U treatment. No significant differences were observed among treatments for spikelets per panicle, seed setting rate, or grain weight in either cultivar. In 2023, the effective panicle number in both cultivars followed the order A > U > N, and the A treatment significantly increased panicle number by 81.32% to 106.05% in X13 and 66.67–87.53% in YZX relative to the U treatment. In addition, for X13, seed setting rate and grain weight under N and A treatments were significantly reduced by 49.59% to 92.42% and 4.97% to 8.08%, respectively, compared with the U treatment. For YZX, spikelets per panicle under the A treatment were significantly lower than those under the U and N treatments by 148.61% to154.69%, whereas the seed setting rate was significantly higher than that under the U treatment by 36.80%, and grain weight was significantly higher than that under the N treatment by 5.71%.

Under field conditions, nitrogen fertilizer formulation also had a dominant effect on effective panicle number, although cultivar-specific responses were evident (Figure 2B,D,F,H). In 2022, the effective panicle number of X13 under the N treatment was significantly lower than that under the U and A treatments by 32.88% and 41.78%, respectively. By contrast, for YZX, the effective panicle number under the A treatment was significantly higher than that under the U and N treatments by 24.45% and 38.33%, respectively. Spikelets per panicle, seed setting rate, and grain weight in X13 showed no significant treatment effects; however, grain weight in YZX under A was significantly higher than that under the N treatment by 5.66%. In 2023, the effective panicle number of X13 under N and A was significantly higher than that under U by 33.62% and 46.55%, respectively, whereas the seed setting rate under N was significantly reduced by 38.71% and 26.36% compared with U and A, and grain weight under N and A decreased by 8.08% and 4.97% relative to U. For YZX, spikelets per panicle under A were significantly higher than those under U and N by 93.05% and 67.69%, but the seed setting rate showed the opposite pattern, decreasing by 37.89% and 35.64%, respectively. In addition, grain weight under N was significantly lower than that under U and A by 4.46% and 5.71%.

### 2.3. Effects of Nitrogen Fertilizer Formulation on Cd Distribution and Translocation in Rice

**Figure 3:**
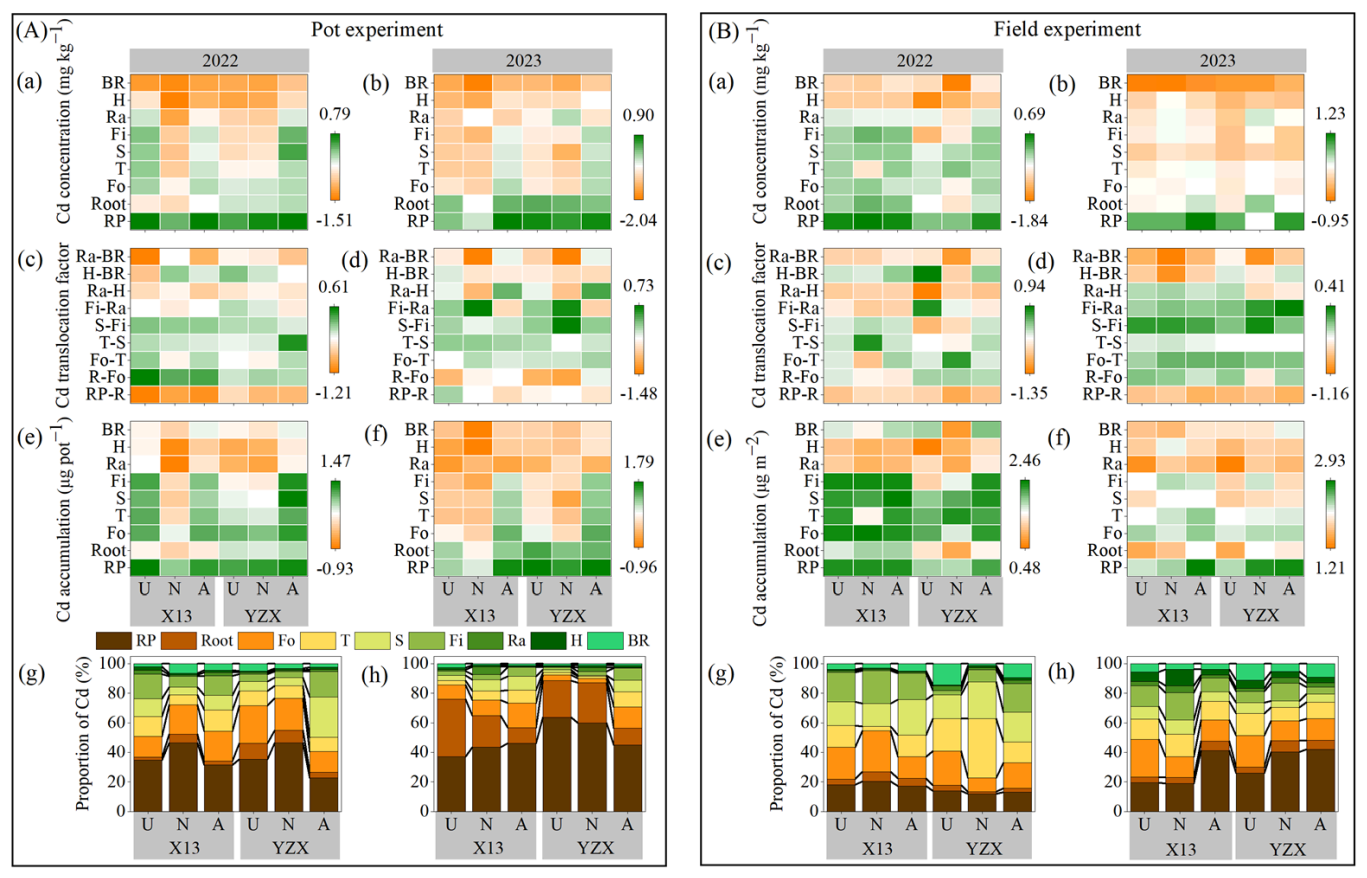
Effects of nitrogen fertilizer formulations on Cd concentrations, translocation factors, accumulation, and distribution proportions in different rice organs under pot and field conditions. Cd concentrations and translocation factors were log_10_-transformed. Figures (**A**) and (**B**) indicate pot and field conditions, respectively; Figures (**a**,**b**) show Cd concentrations in different rice organs in 2022 and 2023, respectively; Figures (**c**,**d**) present the corresponding Cd translocation factors in 2022 and 2023, respectively; Figures (**e**,**f**) show Cd accumulation in 2022 and 2023, respectively; and Figures (**g**,**h**) show Cd proportions in 2022 and 2023, respectively. RP, iron plaque; R, root; Fo, fourth basal stem section; T, third basal stem section; S, second basal stem section; Fi, first basal stem section; Ra, rachis; H, husk; BR, brown rice; U, urea; N, nitrate-N; A, ammonium-N. X13, Xiangwanxian 13 hao; YZX, Yuzhenxiang. Color intensity indicates the relative magnitude, with darker green and orange representing higher and lower values, respectively.

The Cd distribution pattern within rice plants was generally consistent across the pot (Figure 3A(a,b)) and field (Figure 3B(a,b)) experiments. Cd concentration was highest in the iron plaque (RP) and roots, intermediate in the basal stem sections (Fo, T, S, Fi), and lowest in the rachis (Ra), husk (H), and brown rice (BR). One notable exception occurred in 2022 for cultivar X13 under the U and A treatments, where Cd concentrations in the stem sections from Fo to Fi were higher than those in the root. Across all tested conditions, the N treatment consistently produced the lowest Cd concentrations in all plant parts, whereas the A treatment resulted in the highest concentrations. The U treatment generally showed intermediate Cd concentrations.

In the pot experiments, the translocation factor from RP to roots (TF_RP–R) was generally low. In comparison, translocation between aboveground sections was more variable and was strongly affected by nitrogen fertilizer formulation, especially along the pathway from Fo to Ra (Figure 3A(c,d)). Clear treatment-related differences were observed at specific translocation steps. For example, TF_Fo–T in Xiangwanxian 13 hao (X13) in 2022, TF_T–S in Yuzhenxiang (YZX) in 2022, and both TF_S–Fi and TF_Fi–Ra for the two cultivars in 2023 were significantly affected by nitrogen fertilizer formulation.

The field experiments showed greater variability in translocation factors between Fo and Ra than the pot experiments (Figure 3B(c,d)). In 2022, both TF_Fo–T and TF_T–S showed significant treatment differences in the two cultivars (Figure 3B(c)). In 2023, translocation activity, indicated by TF_S–Fi and TF_Fi–Ra, was higher than that in the other stem sections for both cultivars (Figure 3B(d)). Detailed ANOVA results for Cd concentrations and translocation factors across years, cultivars, and environments are presented in Supplementary Tables S1–S6, respectively.

To assess the overall efficiency of Cd transport from roots to aboveground parts, the root-to-aboveground parts translocation factor (TF_Root–AP) was calculated on a flux basis as the ratio of total Cd accumulation in aboveground parts to that in roots. Multifactor ANOVA showed that the main effect of nitrogen fertilizer formulation on TF_Root–AP was not significant, whereas significant effects were detected for cultivar, year, cultivation system, and their interactions with nitrogen fertilizer formulation (Table S6). Under pot conditions, YZX consistently showed higher TF_Root–AP values under A than under U and N treatments, while the ranking for X13 differed between years (Table S4). Under field conditions, no consistent effect of nitrogen fertilizer formulation was observed across years for either cultivar (Table S5). Taken together, these findings indicate that Cd translocation from roots to aboveground parts is modulated by nitrogen fertilizer formulation, but its direction and magnitude are strongly context-dependent and vary with cultivar, year, and cultivation system.

Under both pot and field conditions, the trend in Cd accumulation in different plant parts under different nitrogen fertilizer treatments was generally consistent with the trend in Cd concentration (Figure 3A(e,f),B(e,f)).

Nitrogen fertilizer treatments significantly affected the proportion of Cd in different plant parts, and these effects also varied considerably between cultivation systems (Figure 3A(g,h),B(g,h)). Under pot conditions in 2022, the Cd proportions in different parts of X13 and YZX were markedly changed under the N and A treatments, respectively, compared with the other treatments (Figure 3A(g)). Specifically, in X13 under the N treatment, the Cd proportions in RP, R, and BR increased significantly, whereas those in T, S, Fi, and H decreased significantly. For YZX under the A treatment, the Cd proportions in RP, R, and Fo decreased, while those in S and Fi increased. In 2023, for X13, the Cd proportions in Root and T changed significantly in response to the N treatment. In addition, compared with the N and A treatments, the U treatment significantly reduced the Cd proportions in T, S, and Fi. For YZX under the A treatment, the Cd proportions in RP and Root were significantly lower than those under the U and N treatments, whereas those in Fo, T, S, and Fi increased significantly (Figure 3A(h)).

Under field conditions in 2022, the N treatment significantly increased the Cd proportion in Fo of X13, while significantly decreasing that in T. For YZX, the Cd proportions in Fo, T, S, and BR were significantly affected by nitrogen treatments. Specifically, under the N treatment, the Cd proportions in T and S increased significantly, whereas those in Fo, Ra, and BR decreased significantly (Figure 3B(g)). In 2023, for X13, the Cd proportions in different parts under the A treatment showed greater changes than those under the other treatments: the Cd proportions in RP and Root increased significantly, whereas those in S, Fi, and H decreased significantly. For YZX, under the U treatment, the Cd proportions in RP, Root, and Ra decreased significantly, while those in Fo, T, S, H, and BR increased significantly (Figure 3B(h)). Detailed ANOVA results for Cd accumulation and Cd proportions across years, cultivars, and environments are provided in Supplementary Tables S7–S12, respectively.

### 2.4. Effects of Nitrogen Fertilizer Formulation on Fe Concentration and Cd/Fe Ratio in Iron Plaque

**Figure 4:**
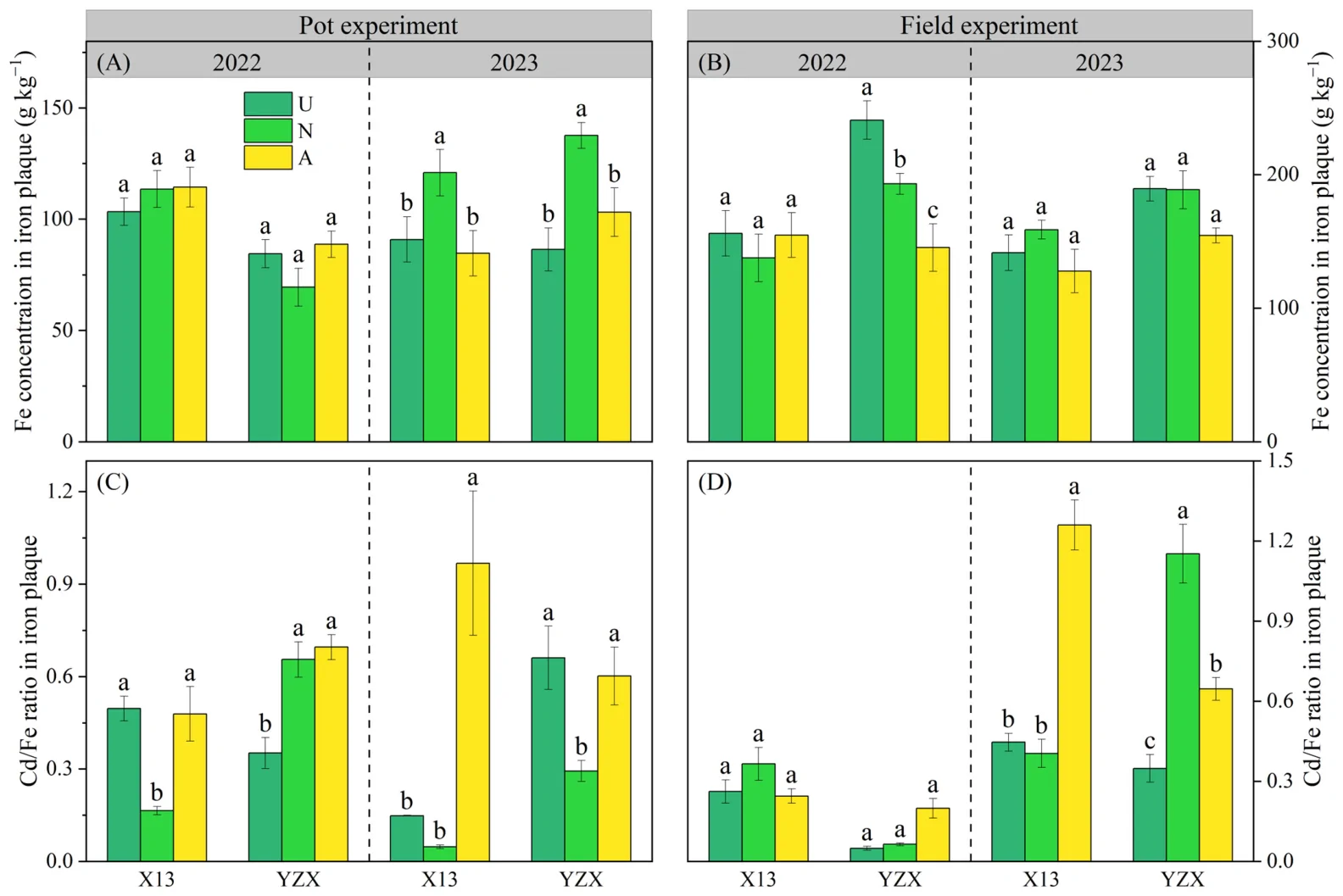
Effects of different nitrogen fertilizer formulations on Fe concentration and Cd/Fe ratio in iron plaque under pot and field conditions. (**A**) Fe concentration in iron plaque under pot conditions (2022–2023); (**B**) Fe concentration in iron plaque under field conditions (2022–2023); (**C**) Cd/Fe ratio in iron plaque under pot conditions (2022–2023); (**D**) Cd/Fe ratio in iron plaque under field conditions (2022–2023). U, urea; N, nitrate-N; A, ammonium-N. X13, Xiangwanxian 13 hao; YZX, Yuzhenxiang. Bars indicate means ± standard error (SE; *n* = 3). Different lowercase letters above the bars indicate significant differences among nitrogen treatments within each cultivar and cultivation condition (Tukey’s test, *p* < 0.05).

In the pot trials, Fe concentration in the iron plaque showed no significant differences among the three nitrogen treatments for either cultivar in 2022. In 2023, however, the nitrate-N (N) treatment significantly increased Fe concentration compared with the urea (U) and ammonium-N (A) treatments, with increases of 33.03–42.80% for Xiangwanxian 13 hao (X13) and 33.42% to 59.25% for Yuzhenxiang (YZX) (Figure 4A).

Under field conditions, Fe concentration in the iron plaque of X13 did not differ significantly among treatments in 2022. For YZX, however, a clear ranking of U > N > A was observed, with the U treatment being 24.69% and 65.72% higher than the N and A treatments, respectively. In 2023, no significant differences were detected among treatments for either cultivar (Figure 4B).

In the pot experiment, the Cd/Fe ratio of X13 under the N treatment in 2022 was significantly lower than those under the U and A treatments. In YZX, the Cd/Fe ratio under the U treatment was significantly lower than that under the N and A treatments. In 2023, the Cd/Fe ratio of X13 under the A treatment was significantly higher than those under the U and N treatments, whereas in YZX, the Cd/Fe ratio under the N treatment was significantly lower than those under the U and A treatments (Figure 4C).

By contrast, under field conditions in 2022, no significant differences in the Cd/Fe ratio were observed among treatments for either cultivar. In 2023, the Cd/Fe ratio of X13 under the A treatment was higher than those under the U and N treatments. In YZX, the N and A treatments increased the Cd/Fe ratio compared with the U treatment (Figure 4D).

### 2.5. Contribution of Plant Cd Variables to Brown Rice Cd Accumulation (RF Analysis)

**Figure 5:**
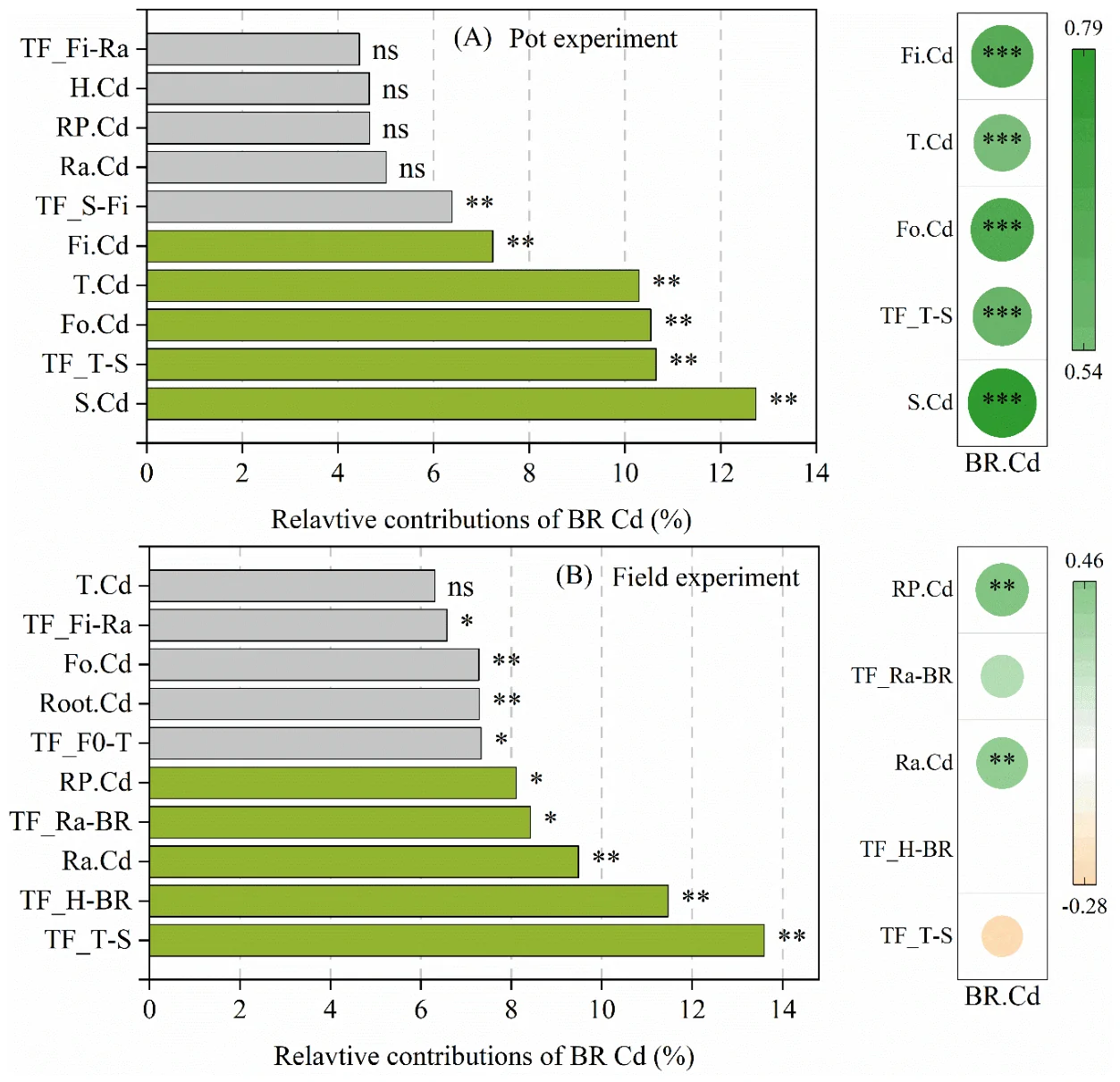
Random forest (RF) analysis identifying the 10 most important factors associated with Cd concentration in brown rice (BR) under different cultivation conditions. Results are shown for the pot (**A**) and field (**B**) experiments. RP, iron plaque; Fo, fourth basal stem section; T, third basal stem section; S, second basal stem section; Fi, first basal stem section; Ra, rachis; H, husk; BR, brown rice; TF, translocation factor. *, **, ***, and ns indicate significance at *p* < 0.05, *p* < 0.01, *p* < 0.001, and *p* ≥ 0.05, respectively. Green and orange indicate positive and negative correlations, respectively, and color intensity represents correlation strength.

Random forest (RF) analysis was applied as an exploratory method to screen for plant variables associated with Cd accumulation in brown rice (BR). In the pot experiment, the top 10 contributing factors were mainly associated with Cd concentrations in basal organs and Cd translocation toward upper plant parts (Figure 5A). The five most important factors included Cd concentration in the S section (S.Cd), the translocation factor from T to S (TF_T–S), and Cd concentrations in Fo, T, and Fi. All these variables were significantly and positively correlated with BR Cd concentrations. The remaining factors, ranked 6–10, included the translocation factor TF_S–Fi, Cd concentrations in Ra, RP, and H, and TF_Fi–Ra.

In the field experiment, the key factors were more diverse and included Cd concentrations in root and basal tissues, Cd concentrations in middle and upper plant parts, and translocation factors involving these tissues (Figure 5B). The five leading factors were TF_T–S, TF_H–BR, Cd concentration in Ra, TF_Ra–BR, and Cd concentration in RP. Among these variables, Cd concentrations in RP and Ra were significantly and positively correlated with BR Cd concentration. Other significant contributors included Cd concentrations in roots and Fo, as well as the translocation factors TF_Fo–T and TF_Fi–Ra. The effects of year, cultivar, cultivation system, and their interactions with nitrogen fertilizer formulation on the relevant Cd concentrations and translocation factors were evaluated by multifactor ANOVA (Supplementary Tables S3 and S6). It should be emphasized that RF analysis provides a correlative ranking of variable importance and does not establish causation. Therefore, the RF results were interpreted as an exploratory approach for identifying candidate variables for subsequent statistical evaluation, rather than as direct evidence of causal regulation.

### 2.6. ANOVA of Key Factors Influencing Brown Rice Cd Concentration

**Figure 6:**
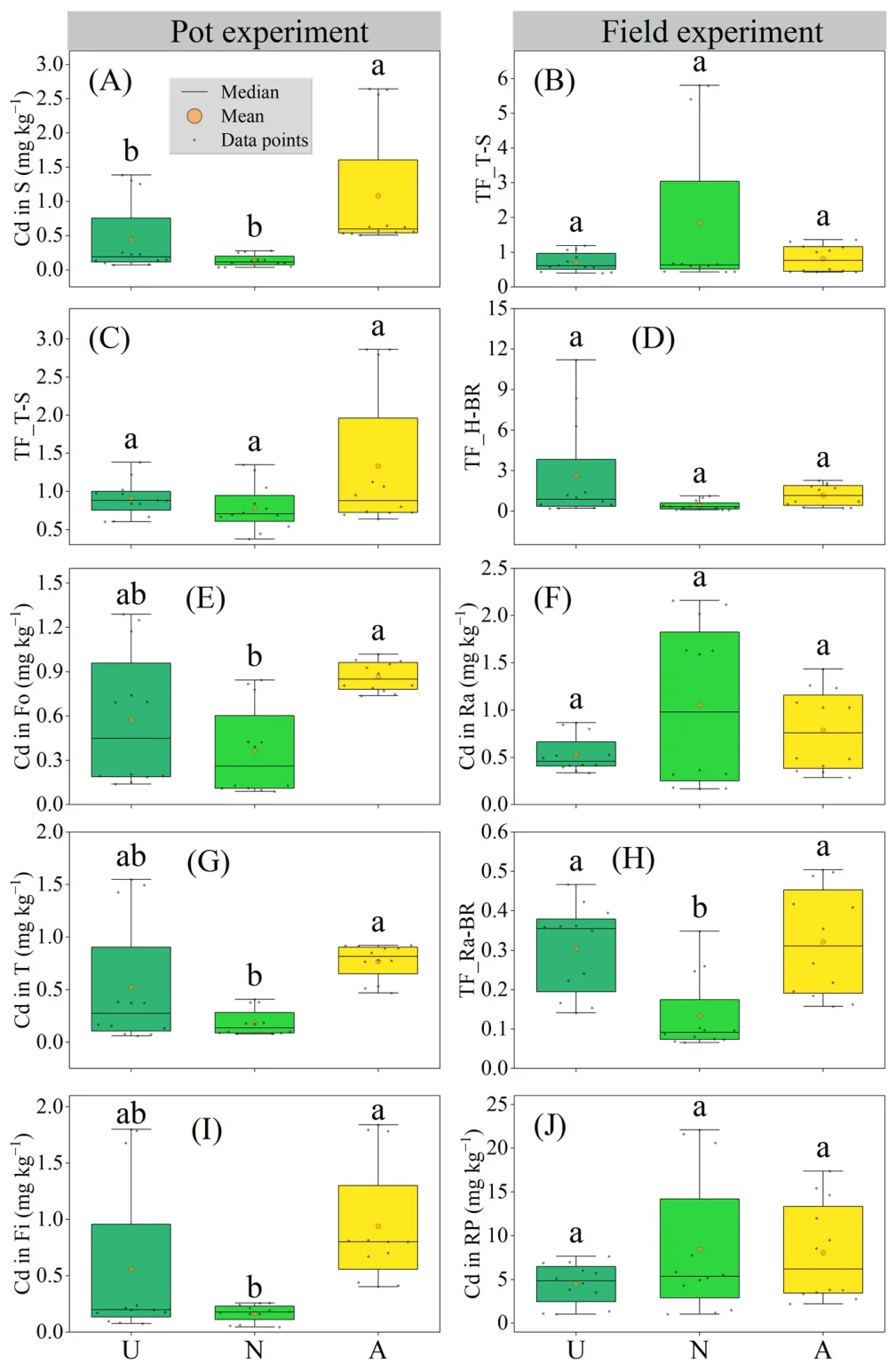
Analysis of variance (ANOVA) for key factors influencing brown rice (BR) Cd concentration under pot and field conditions across two years. (**A**,**C**,**E**,**G**,**I**) Key Cd-related traits under pot conditions: Cd concentration in the S internode (**A**), Cd concentration in the Fo internode (**C**), Cd concentration in the T internode (**E**), Cd concentration in the Fi internode (**G**), and translocation factor TF_T–S (**I**). (**B**,**D**,**F**,**H**,**J**) Corresponding traits under field conditions: TF_T–S (**B**), TF_H–BR (**D**), Cd concentration in the rachis (Ra, (**F**)), TF_Ra–BR (**H**), and Cd concentration in the iron plaque (RP, (**J**)). RP, iron plaque; Fo, fourth basal stem section; T, third basal stem section; S, second basal stem section; Fi, first basal stem section; Ra, rachis; H, husk; BR, brown rice; TF, translocation factor. U, urea; N, nitrate-N; A, ammonium-N. Different lowercase letters above the bars indicate significant differences among nitrogen treatments (Tukey’s test, *p* < 0.05).

In the pot experiment, Cd concentration in the S section was significantly higher under the A treatment than under the U and N treatments (Figure 6A). Similarly, nitrogen fertilizer formulation significantly affected Cd concentrations in the Fo, T, and Fi sections (Figure 6C,E,G). In these internodes, the A treatment resulted in significantly higher Cd concentrations than the N treatment, whereas no significant difference was found between the A and U treatments. By contrast, the translocation factor from T to S (TF_T–S) was not significantly affected by nitrogen fertilizer formulation in the pot experiment (Figure 6B).

In the field experiment, the translocation factor from Ra to BR (TF_Ra–BR) was significantly lower under the N treatment than under the U and A treatments (Figure 6H). However, Cd concentrations in Ra and RP, as well as the translocation factors TF_T–S and TF_H–BR, did not differ significantly among the nitrogen fertilizer treatments (Figure 6B,D,F,J).

The high importance of TF_T–S in the RF ranking (Figure 5A), despite its nonsignificant ANOVA result (Figure 6B), may reflect collinearity between TF_T–S and other Cd-related variables, such as Cd concentrations in adjacent stem sections, rather than a direct regulatory effect of nitrogen fertilizer formulation on this specific translocation step. This inconsistency indicates that RF importance ranking should be interpreted as an exploratory association measure rather than a confirmatory test of treatment effects.

### 2.7. Effects of Nitrogen Fertilizer Formulation on Rhizosphere Soil Properties in the Pot Experiment

**Figure 7:**
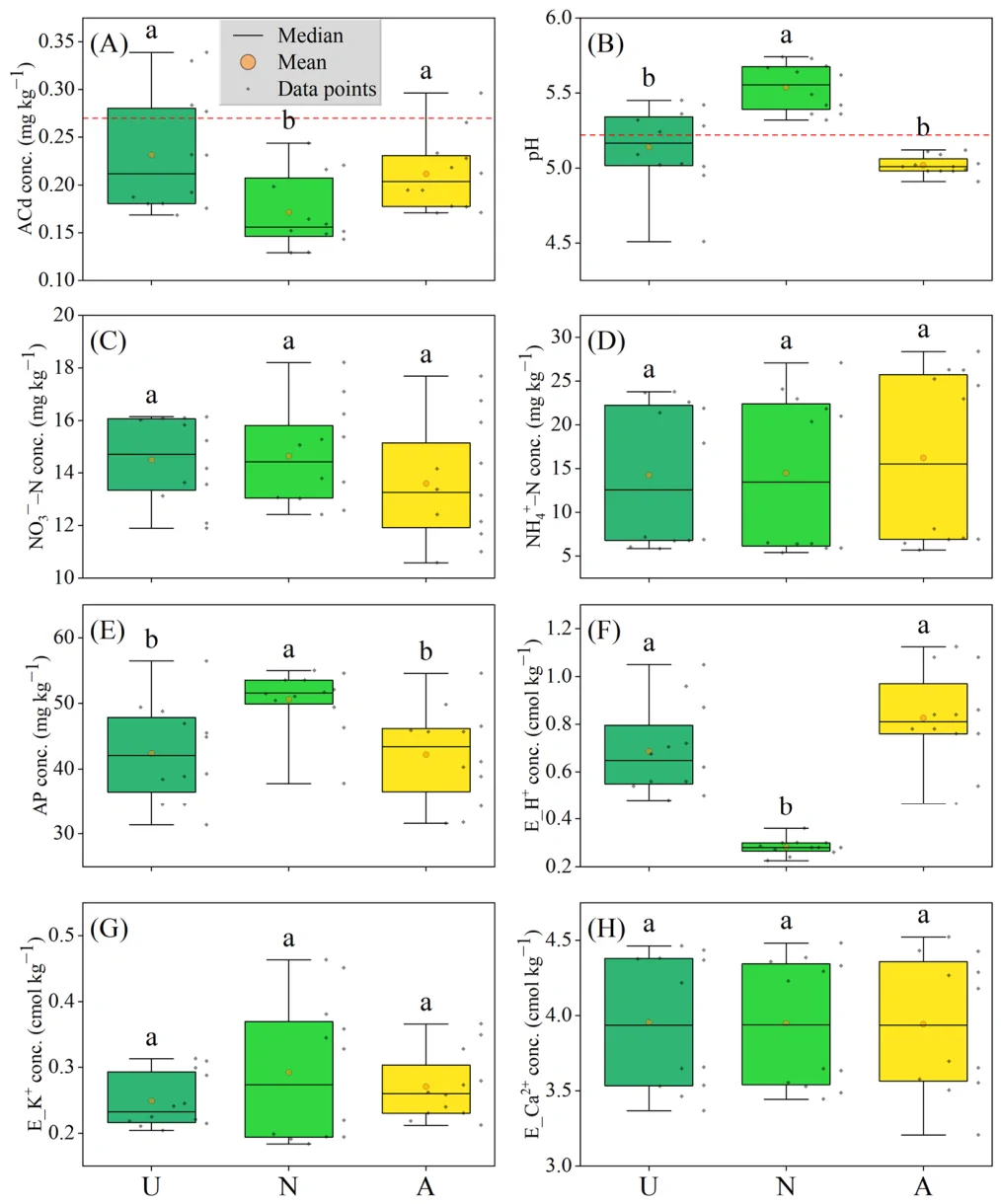
Analysis of variance (ANOVA) for the effects of nitrogen fertilizer formulations on rhizosphere soil properties under pot conditions across two years. The measured properties include available Cd (ACd, (**A**)), pH (**B**), NO_3_^–^–N (**C**), NH_4_^+^–N (**D**), available phosphorus (AP, (**E**)), exchangeable H^+^ (E_H^+^, (**F**)), exchangeable K^+^ (E_K^+^, (**G**)), and exchangeable Ca^2+^ (E_Ca^2+^, (**H**)). U, urea; N, nitrate-N; A, ammonium-N. Different lowercase letters indicate significant differences among nitrogen treatments (*p* < 0.05). The red dashed line indicates the initial soil pH and available Cd concentration.

To evaluate the overall effect of nitrogen fertilizer formulation on the rhizosphere environment, data from both years and cultivars were pooled for analysis, while year- and cultivar-specific variations are shown in Figure S1. Nitrogen fertilizer formulation significantly altered several key rhizosphere properties (Figure 7). Compared with the N treatment, the U and A treatments significantly increased the concentrations of available Cd (ACd; Figure 7A) and exchangeable H^+^ (E_H^+^; Figure 7F), while significantly decreasing soil pH (Figure 7B) and available phosphorus (AP; Figure 7E). However, no statistically significant differences were observed between the U and A treatments for any of these variables. In addition, concentrations of soil NO_3_^–^–N, NH_4_^+^–N, exchangeable K^+^ (E_K^+^), and exchangeable Ca^2+^ (E_Ca^2+^) did not differ significantly among the three nitrogen fertilizer treatments (Figure 7C,D,G,H).

### 2.8. Mantel Correlation Between Rhizosphere Soil Factors and Key Plant Cd Factors in the Pot Experiment

**Figure 8:**
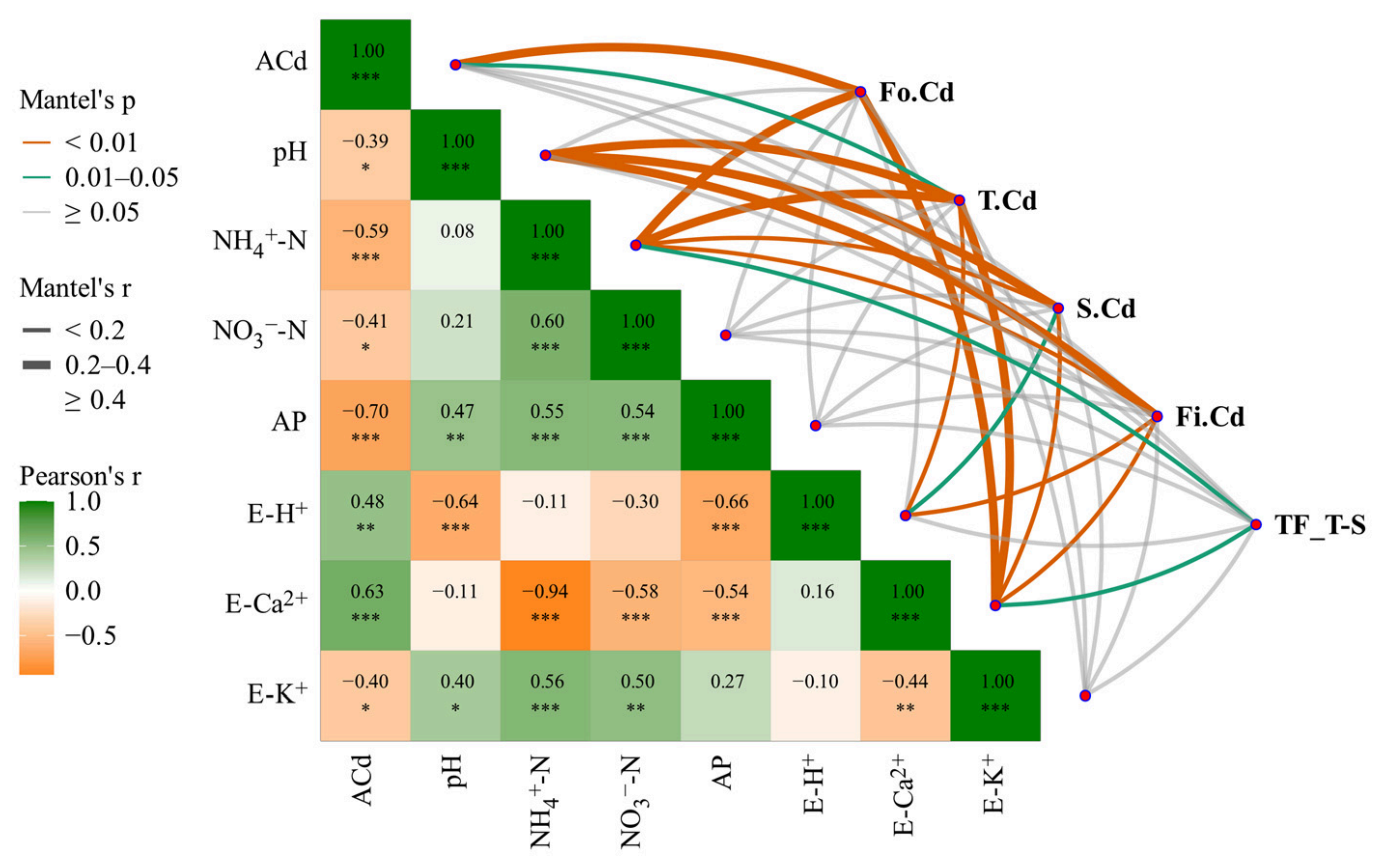
Mantel correlations between rhizosphere soil environmental factors and the top five Cd-related plant factors under pot conditions. ACd, soil available Cd; pH, soil pH; NO_3_^–^–N, soil nitrate nitrogen; NH_4_^+^–N, soil ammonium nitrogen; AP, available phosphorus; E_H^+^, exchangeable H^+^; E_K^+^, exchangeable K^+^; E_Ca^2+^, exchangeable Ca^2+^. Fo.Cd, T.Cd, S.Cd, and Fi.Cd represent Cd concentrations in the fourth, third, second, and first basal stem sections, respectively; TF_T-S represents the Cd translocation factor from T to S. Green and orange represent positive and negative correlations, respectively. Color intensity corresponds to the strength of the correlation. *, **, and *** indicate significance at *p* < 0.05, 0.01, and 0.001, respectively.

To examine the linkages between the rhizosphere environment and the key processes of Cd accumulation and translocation identified in the pot experiment, Mantel correlation analysis was performed using the pooled dataset (Figure 8).

The analysis revealed a network of significant correlations among soil properties. Rhizosphere soil ACd concentration was significantly and negatively correlated with soil pH, NH_4_^+^–N, NO_3_^–^–N, and E_K^+^, but was significantly and positively correlated with E_H^+^ and E_Ca^2+^. Soil pH was negatively correlated with E_K^+^. Both NH_4_^+^–N and NO_3_^–^–N were negatively correlated with E_Ca^2+^. AP was negatively correlated with E_H^+^ and E_Ca^2+^. By contrast, AP and E_K^+^ were positively correlated with pH, NH_4_^+^–N, and NO_3_^–^–N. A positive correlation was also found between NH_4_^+^–N and NO_3_^–^–N.

Significant correlations were also identified between specific soil factors and key plant variables. Soil ACd was significantly correlated with Cd concentrations in Fo and T. Soil pH and E_H^+^ were correlated with Cd concentrations in T, S, and Fi. In addition, NH_4_^+^–N and E_Ca^2+^ were correlated with Cd concentrations across all four measured basal internodes (Fo, T, S, and Fi).

## 3. Discussion

### 3.1. A Stable Trade-Off: Opposite Effects of Nitrogen Fertilizer Formulations on Rice Yield and Grain Cd Accumulation

Our two-year study, integrating both pot and field systems, consistently demonstrated a trade-off between rice grain yield and Cd accumulation that was governed by nitrogen fertilizer formulation. Nitrate-N (N) consistently reduced Cd concentration in BR, but this reduction occurred at the expense of yield, whereas ammonium-N (A) maintained or increased yield while significantly enhancing grain Cd accumulation. Urea (U) showed an intermediate response (Figure 1). This clear inverse relationship, observed across cultivation systems, years, and cultivars, suggests an inherent physiological response to nitrogen fertilizer formulation rather than an experimental artifact. This finding is consistent with previous reports showing that nitrate can suppress Cd translocation to grains but may limit biomass, whereas ammonium, the preferred nitrogen source for rice, supports yield but often promotes Cd uptake and mobility [18]. Our multi-environment results further confirmed the stability of this trade-off.

The magnitude of the yield–Cd trade-off varied considerably among treatments and systems. In this study, compared with the U treatment, the N treatment reduced brown rice Cd by 4.25% to 89.97% but decreased yield by 5% to 25%, whereas the A treatment increased grain Cd by 17.86% to 58.62% with a yield change of –3% to +5%. This broad range indicates that cultivar, year, and cultivation system influenced the strength of the trade-off. For example, the exceptionally high Cd reduction of nearly 90% observed in Yuzhenxiang (YZX) under the N treatment in 2022 (Figure 1D) suggests that certain genotype-by-environment combinations may amplify the Cd-mitigating effect of nitrate. This outlier may be associated with the combined effects of genotype-specific sensitivity and interannual environmental variation: YZX is an aromatic rice cultivar with distinct physiological characteristics, and climatic conditions during the 2022 growing season, particularly around the heading stage, differed from those in 2023. Previous studies have shown that temperature and water availability can affect nitrate reductase activity and nitrogen metabolism in rice [23], which may further influence Cd partitioning. However, because detailed meteorological data were not recorded in the present study, this explanation remains speculative. Although such extreme responses may not be generalizable across all environments, the consistent qualitative pattern, namely that the N treatment reduced Cd with a yield cost and the A treatment maintained yield while increasing Cd, remained robust across the other cultivar-year combinations. This indicates that although the precise magnitude of the trade-off is context-dependent, its direction is stable, supporting the development of nitrogen management strategies tailored to specific soil Cd levels and production objectives.

The yield penalty under nitrate-N requires particular attention because it directly affects the practical feasibility of nitrate-based Cd mitigation. Rice grown under flooded paddy conditions is generally adapted to ammonium-dominant nutrition. Compared with ammonium-N, nitrate-N may be used less efficiently under submerged conditions because of lower physiological preference, possible nitrogen transformation losses, and weaker compatibility with root metabolic traits adapted to reduced soils [24,25]. These factors may restrict tillering, biomass accumulation, panicle formation, and final grain yield. Therefore, although nitrate-N reduced BR Cd, its agronomic value should be evaluated together with its potential cost to productivity.

Notably, our observation that ammonium promoted Cd accumulation in rice differs from some phytoremediation studies in which nitrate enhanced Cd extraction in species such as sweet sorghum [26] and Bidens pilosa [27]. This inconsistency emphasizes the strong species-specific nature of plant responses to nitrogen. As an ammonium-preferring crop, rice likely has a distinct Cd regulatory pathway under ammonium nutrition, involving rhizosphere processes and internal transport mechanisms that differ from those in nitrate-preferring species. Therefore, nitrogen management strategies for HM mitigation should be adapted to the nutritional physiology of the target crop.

Nevertheless, the possibility that accompanying ions (Ca^2+^ and SO_4_^2–^) contributed to the observed responses should be considered when interpreting these results. The absence of ion-balanced controls, such as CaCl_2_ or K_2_SO_4_ supplementation, prevents complete separation of nitrogen fertilizer formulation effects from counterion effects.

### 3.2. Mechanisms of Coordinated Regulation: From Rhizosphere Chemical Processes to Internal Plant Translocation Nodes

Analysis under controlled pot conditions showed that nitrogen fertilizer formulations significantly changed key rhizosphere properties, including soil pH, E_H^+^, and ACd concentration (Figure 7A,B,F). Specifically, the A treatment decreased rhizosphere pH and increased ACd, whereas the N treatment was associated with relatively higher pH and lower ACd concentrations. Mantel correlation analysis further confirmed a significant negative correlation between ACd and pH and a positive correlation between ACd and E_H^+^ (Figure 8). However, it should be emphasized that the pH-centered mechanism proposed in this study represents only one component of a more complex biogeochemical system. In flooded paddy soils, redox-driven processes, including Fe(III) reduction, sulfate reduction, and dissolved organic carbon (DOC)-mediated complexation, are often equally or more important than pH changes in controlling Cd mobility [28,29]. Although these processes could not be quantified because Eh, Fe^2+^, Mn^2+^, and DOC were not measured, their potential contributions and interactions with the observed pH effects must be carefully considered when interpreting the results.

It should also be noted that the nitrate and ammonium treatments were represented by calcium nitrate and ammonium sulfate, respectively. Therefore, Ca^2+^ and SO_4_^2−^ may have contributed to the treatment effects by modifying ionic strength, Cd adsorption, or nutrient interactions. Although equivalent nitrogen rates were applied, the lack of ion-balanced controls prevents complete separation of nitrogen-form effects from counterion effects. Future experiments including CaCl_2_ or K_2_SO_4_ balanced treatments would help strengthen causal interpretation.

These results indicate that nitrogen fertilizer treatments were associated with changes in the measured rhizosphere chemical properties, particularly pH, exchangeable H^+^, and available Cd. Because redox-sensitive variables, including soil Eh, Fe^2+^, Mn^2+^, and DOC, were not measured, the contribution of redox-driven processes to Cd mobilization could not be quantified. Therefore, the present interpretation should be regarded as a pH-centered mechanism supported by the measured variables, rather than a complete description of Cd biogeochemical regulation in flooded paddy soil. We propose an integrated “rhizosphere regulation pathway” to explain these patterns. Although not directly measured here, previous studies suggest that: (1) proton release during ammonium uptake acidifies the rhizosphere and promotes the desorption of soil-sorbed Cd. Meanwhile, acidification is often accompanied by increased root exudation of organic acids, which can chelate Cd and enhance its solubility [18,30]. Mechanistic studies further suggest that ammonium assimilation can upregulate genes, including members of the *ALMT* family, thereby promoting organic acid efflux [31], which provides a possible genetic basis for enhanced chelation in our system. (2) The combined effects of acidification and organic inputs may reshape the rhizosphere microbiome, potentially enriching bacterial taxa such as *Proteobacteria* and functional genes that jointly enhance Cd mobilization [14,24].

In flooded paddy soils, Cd availability is also strongly influenced by redox potential, Fe/Mn reduction, iron plaque dynamics, and DOC [7,22]. Because Eh, Fe^2+^, Mn^2+^, and DOC were not measured in this study, the contribution of redox-driven processes to Cd mobilization could not be directly assessed. Several redox-related processes may counteract or interfere with the pH effects observed in this study. First, the reductive dissolution of Fe(III) (hydr)oxides under anaerobic conditions can release Cd that was previously adsorbed onto iron oxide surfaces, potentially overriding pH-induced immobilization [28]. Second, under strongly reducing conditions, sulfate reduction can lead to the Cd precipitation as CdS, a process largely controlled by sulfide availability, which may reduce Cd solubility largely independently of pH [29,32]. Third, DOC-mediated complexation, which often increases under reducing conditions because of enhanced organic matter decomposition, can solubilize Cd and facilitate its transport even under neutral or alkaline pH conditions [2]. These processes may act independently or jointly, and their relative importance is likely to vary with flooding duration and intensity, soil organic matter content, and sulfur availability. Therefore, the observed pH effects should be interpreted as part of an integrated system, and the absence of redox data represents a major limitation in the mechanistic interpretation.

In contrast, nitrate uptake generally alkalizes the rhizosphere and has weaker effects on root exudation and microbial activity [33], thereby limiting the mobilization of fixed Cd [18,30]. The influence of nitrogen on Cd uptake is highly context-dependent. For example, in pH-buffered hydroponic systems, ammonium can suppress Cd uptake by downregulating transporters such as *OsIRT1* and *OsNRAMP5* [10]. This contrast indicates that in our soil system, ammonium-induced acidification and the resulting Cd activation were the dominant drivers of higher plant Cd, outweighing any potential direct inhibitory effects of ammonium on uptake transporters. The strong association between the A treatment, decreased pH, and increased E_H^+^ (Figure 7B,F) further supports the conclusion that altered rhizosphere conditions represent an important link between nitrogen fertilizer formulation and plant Cd acquisition.

Across both pot and field conditions, Cd showed a consistent distribution pattern, with the highest concentrations in roots and basal stem segments (Fo, T, S, Fi) and the lowest in Ra, H, and BR (Figure 3A,B). This pattern is consistent with the “root-stem barrier-panicle redistribution” theory [34] and supports our second hypothesis that grain Cd is controlled not only by total uptake but also by specific internal allocation processes. Roots and iron plaques function as primary filters [35,36], whereas basal stem segments may act as temporary sequestration sites, as indicated by Cd concentrations that occasionally exceeded those in roots. Subcellular sequestration, such as sequestration in cell walls, forms an important Cd “pool” [15], and ammonium can affect the subcellular distribution and speciation of Cd, thereby affecting inter-organ translocation [37]. Thus, Cd distribution probably reflects a dynamic balance between sequestration and redistribution.

The final allocation of Cd to grains depends on the efficiency with which Cd is redistributed within stems and panicles [12]. Ammonium-N promoted this redistribution more strongly than nitrate-N (Figure 3A,B), which agrees with previous findings [18]. To identify the major controlling steps, translocation factor analysis was combined with random forest modeling. The RF screening showed that translocation parameters, including TF_T–S, TF_Ra–BR, and TF_H–BR, were more strongly associated with BR Cd than Cd concentrations in individual organs (Figure 3A,B), indicating that Cd redistribution among organs may be particularly important for grain Cd accumulation. Notably, the high RF importance of TF_T–S in the pot experiment (Figure 5A) was not supported by ANOVA (Figure 6B), suggesting that this variable may be associated with BR Cd through collinearity with other stem Cd concentrations rather than through direct regulation by nitrogen fertilizer formulation. The translocation steps identified in this study are consistent with previously reported Cd transport pathways in rice. For example, *OsHMA2* has been implicated in root-to-shoot and node-mediated Cd transport [10], whereas *OsLCT1* participates in Cd transfer to grains [37]. These transporters may therefore represent candidate molecular components related to the nitrogen-formulation-associated translocation patterns observed here. Wu et al. [21] reported that NO_3_^–^ treatment upregulated *OsIRT1* and *OsHMA2* expression in rice roots, whereas NH_4_^+^ increased *OsNRAMP5* expression, demonstrating transcriptional regulation of key transporters by nitrogen form. Consistent with these findings, our study showed that ammonium treatment increased Cd levels at key transport nodes in YZX (Tables S1, S2, S4 and S5) and increased the root-to-shoot translocation factor (TF_Root–AP; Table S4), suggesting that ammonium may enhance both root uptake through *OsNRAMP5* and shoot translocation through *OsHMA2*/*OsLCT1*. Furthermore, the normalized Cd/Fe ratio in iron plaque did not differ among nitrogen treatments (Figure 4A,B), indicating that ammonium-enhanced Cd accumulation was mainly driven by increased plaque biomass rather than enhanced adsorption efficiency. Nevertheless, it is important to recognize that the absence of redox-related measurements, including Eh, Fe^2+^, Mn^2+^, and DOC, places important constraints on the interpretation of our results. Although our data provide strong evidence for nitrogen-formulation-associated changes in pH, exchangeable H^+^, and available Cd, the relative importance of pH and redox mechanisms in Cd mobilization cannot be determined from the present dataset. The observed cultivar-and year-specific variations in Cd accumulation may partly reflect redox-related differences in Fe/Mn cycling and sulfide precipitation rather than pH effects alone. Consequently, although our findings clearly show that nitrogen fertilizer formulations are associated with changes in the measured rhizosphere chemical properties, the complete causal pathway, particularly the role of redox-mediated processes, remains to be clarified. Future studies combining redox monitoring with the pH-centered approach used here are needed to provide a more comprehensive understanding of Cd behavior in flooded paddy systems [38]. Collectively, these results suggest that ammonium promotes grain Cd accumulation through coordinated regulation of multiple transport nodes, from root uptake to phloem loading, whereas the role of iron plaque remains largely physical.

### 3.3. From Mechanisms to Practice: Nitrogen Management Strategies for Safe Rice Production in Contaminated Paddies

The third hypothesis was further supported because the major regulatory steps controlling grain Cd differed between pot and field systems. Under pot conditions, Cd retention in basal organs and upward movement were more evident, whereas under field conditions, Cd redistribution near the panicle was more important. This difference is probably related to variations in the rhizosphere environment, plant size, and nutrient cycling between the two systems. The 1.5-fold scaling factor used in the pot experiment may have amplified rhizosphere pH shifts, ion accumulation, and Cd availability responses compared with field conditions. This difference in nutrient concentration between the two systems represents an important limitation when pot and field results are directly compared, because the magnitude of treatment effects, rather than the qualitative direction, may differ between systems. Therefore, the quantitative differences between pot and field experiments should not be overinterpreted, and both systems should be considered complementary rather than directly comparable.

This finding has practical implications: screening under controlled conditions, such as pot trials, should focus on root-to-shoot translocation barriers mediated by genes such as *OsHMA3* [14], whereas strategies for field production should target panicle loading mechanisms, including those involving *OsLCT1* [13]. However, because transporter expression was not measured in the present study, these genes should be regarded as candidate targets for future validation rather than mechanisms directly demonstrated here.

Collectively, this study advances the understanding of Cd stress response in rice by clarifying a continuous “rhizosphere–translocation” cascade through which nitrogen fertilizer formulations regulate the yield-Cd trade-off. Unlike previous studies that focused mainly on soil Cd availability or root uptake in isolation, our integrative framework shows that: (i) nitrogen fertilizer formulations modulate Cd bioavailability through rhizosphere pH and exchangeable H^+^; (ii) specific stem internodes, particularly the T–S internode, and panicle-to-grain redistribution, including Ra–BR and H–BR, serve as important nodes controlling final grain Cd; and (iii) the relative importance of these nodes differs between pot and field systems, highlighting the need for system-specific management strategies. By combining rhizosphere chemistry, internal transport analysis, and multi-environment validation, this study provides a more complete mechanistic framework for understanding how agronomic nitrogen management influences Cd accumulation in rice, while also suggesting practical targets, including agronomic approaches such as nitrogen blending and timing and targets such as genetic *OsHMA2* and *OsLCT1* pathways, for developing “stable-yield, low-Cd” production systems in contaminated paddies.

In practice, achieving “stable-yield, low-Cd” production requires systematic strategies rather than single-point interventions. In Cd-contaminated areas, avoiding reliance on a single nitrogen source and using blends, such as urea or nitrate/ammonium mixtures, may help regulate rhizosphere pH. Precise water and fertilizer management at critical growth stages, such as heading, can further limit Cd movement into grains. In addition, the key transport nodes identified in this study provide clear targets for breeding low-Cd rice varieties. Integrating targeted agronomy with genetic improvement may support the development of a multilayered Cd control system from the rhizosphere to the panicle, providing a practical framework for safe rice production in Cd-affected regions.

Despite these implications, several limitations should be acknowledged. First, the experimental design did not include ion-balanced controls, such as CaCl_2_ addition to balance Ca^2+^ or K_2_SO_4_ addition to balance SO_4_^2–^, which limits the complete separation of nitrogen-form effects from counterion effects. Second, redox-related indicators, including Eh, Fe^2+^, Mn^2+^, and dissolved organic carbon, were not measured, which restricts a complete explanation of Cd behavior under flooded conditions. Third, transporter-level molecular evidence was not obtained. Finally, although RF analysis helped identify influential variables, it should be considered an exploratory statistical approach rather than independent evidence of causality. Future research should integrate ion-balanced fertilizer treatments, redox monitoring, and molecular validation of Cd transporter genes to further refine the pathway from nitrogen fertilizer formulation to grain Cd accumulation.

## 4. Materials and Methods

### 4.1. Site and Materials

Field experiments were conducted from 2022 to 2023 in Huayuan Village, Yanxi Town, Liuyang City, Hunan Province, China (28°18′ N, 113°49′ E). In parallel, pot experiments were performed in a rainproof greenhouse at the Rice Research Institute of Hunan Agricultural University (28°18′ N, 113°07′ E) using the same rice cultivars. The field site has a subtropical monsoon humid climate, with a mean annual temperature of 16.8–17.2 °C and annual precipitation of approximately 1400 mm. The soil was classified as a red–yellow clayey paddy soil. The main physicochemical properties of the topsoil (0–20 cm depth) were as follows: total N, 1.48 g kg^–1^; available N, 103.75 mg kg^–1^; available P, 5.97 mg kg^–1^; available K, 129 mg kg^–1^; organic matter, 31.26 g kg^–1^; pH, 5.22; total Cd, 0.80 mg kg^–1^; and available Cd (0.1 M CaCl_2_-extractable), 0.27 mg kg^–1^. Soil for the pot experiment was collected from the same layer (0–20 cm), air-dried, sieved, and thoroughly homogenized before use.

For the field trial, urea (amide-N; 46.6% nitrogen, U), calcium nitrate (nitrate-N; 11.9% nitrogen, N), and ammonium sulfate (ammonium-N; 21.2% nitrogen, A) were used as nitrogen fertilizers representing different nitrogen forms. Monopotassium phosphate (52% P_2_O_5_ and 34% K_2_O) and potassium chloride (60% K_2_O) were used as phosphorus and potassium sources, respectively, and were purchased from local suppliers. Analytical-grade reagents were used as fertilizer sources in the pot experiments (Sinopharm Chemical Reagent Co., Ltd., Shanghai, China). Two locally adapted late-season rice cultivars, Xiangwanxian 13 hao (X13) and Yuzhenxiang (YZX), were tested, and the seeds were provided by Hunan Nongfeng Seed Co., Ltd., Changsha, Hunan, China.

All nitrogen treatments were designed to supply equivalent total nitrogen rates within each experimental system. Therefore, differences among the U, N, and A treatments mainly reflected effects associated with nitrogen form rather than total nitrogen supply. However, the fertilizers used to represent different nitrogen forms also introduced different accompanying ions. Specifically, calcium nitrate supplied Ca^2+^, whereas ammonium sulfate supplied SO_4_^2–^. These accompanying ions may affect Cd adsorption, ionic competition, rhizosphere chemistry, or Cd mobility. Therefore, the present experiment should be interpreted as a comparison of agronomically realistic nitrogen fertilizer formulations rather than a fully ion-balanced single-factor test of nitrate versus ammonium.

It is important to note that the different nitrogen forms were supplied by specific fertilizers: nitrate-N as calcium nitrate with Ca^2+^, ammonium-N as ammonium sulfate with SO_4_^2–^, and urea without a counterion. Ca^2+^ and SO_4_^2–^ may influence Cd adsorption, ionic competition, rhizosphere chemistry, or Cd mobility through their own physicochemical effects. Therefore, the observed differences among treatments cannot be attributed solely to nitrogen form and may also involve accompanying ions. Accordingly, this study is interpreted as a comparison of agronomically realistic nitrogen fertilizer formulations rather than a fully ion-balanced, single-factor test of nitrate versus ammonium.

### 4.2. Experimental Design

#### 4.2.1. Field Experiment

The field experiment was arranged in a randomized complete block design with three replicates per treatment. In this design, urea application (amide-N, abbreviated as U) was used as the control, whereas calcium nitrate (nitrate-N, N) and ammonium sulfate (ammonium-N, A) represented the alternative nitrogen-form treatments. Nitrogen fertilizer management was kept consistent across all plots. The total nitrogen application rate was 180 kg N ha^–1^, with 60% applied as basal fertilizer and 40% applied at the tillering stage. Thus, all field treatments received the same total nitrogen input and the same split-application schedule. Each cultivar was grown separately, resulting in 18 plots in total, each with an area of 48 m^2^. The plots were separated by bunds covered with black plastic film to prevent water and nutrient exchange. Before transplantation, the field was plowed and repeatedly harrowed to homogenize the soil. Rice seeds were sown on June 25 and transplanted on July 25 in 2022, and the corresponding dates in 2023 were June 26 and July 16. The planting density was 16.6 cm × 20.0 cm, with 2–3 seedlings per hill. Phosphorus fertilizer (P) was applied as a basal dose at 90 kg P_2_O_5_ ha^–1^ before transplanting. Potassium (K) was applied at 120 kg K_2_O ha^–1^, split equally between 1 and 2 d before transplanting and the panicle initiation stage. Standard field management practices for irrigation, weeding, and pest control were followed. A shallow water layer of 2–3 cm was maintained throughout the growing period, and irrigation was stopped approximately 10 days before harvest. The same irrigation regime was used for all field treatments to minimize hydrological variation among nitrogen-form treatments.

#### 4.2.2. Pot Experiment

Polyethylene pots with top and bottom diameters of 40 and 35 cm, respectively, and a height of 30 cm were filled with 9 kg of soil. The soil was flooded for 15 days before transplanting and thoroughly mixed to better simulate field conditions. Nitrogen fertilizer application followed the same treatments as those used in the field experiment. Fertilizer rates were calculated based on field application rates and adjusted by increasing them 1.5-fold for pot volume, resulting in applications of 120 mg N, 30 mg P, and 80 mg K per kg of soil. The 1.5-fold scaling factor was used to compensate for restricted rooting volume, lower soil buffering capacity, and potentially faster nutrient depletion under pot conditions, thereby reducing the possibility that rice growth would be limited by insufficient nutrient supply in the confined pot system. Nitrogen was applied with 60% as basal fertilizer and 40% at the tillering stage, whereas potassium was split equally between basal and panicle initiation applications. Phosphorus was applied entirely as basal fertilizer. As in the field experiment, the three nitrogen-form treatments received equivalent total nitrogen inputs and identical nitrogen split timing. Three hills were planted in each pot, with two seedlings per hill. Each treatment included nine replicates, and the cultivars were grown separately, resulting in 54 experimental units in total. Sowing and transplanting dates were synchronized with those of the field experiment (sowing/transplanting: June 25/July 20 in 2022 and June 25/July 15 in 2023). A shallow water layer was maintained throughout the growth period; weeds were removed manually, and pests were controlled as needed. Specifically, the pots were maintained under continuously flooded conditions with a shallow water layer comparable to the field water depth, except during brief management operations. Water was replenished regularly to avoid severe drying, and overflow was prevented to reduce nutrient and solute loss. Although this regime was designed to approximate field flooding management, the small soil volume in pots may have caused stronger fluctuations in rhizosphere solute concentration and redox conditions than those in the field plots. To minimize microenvironmental variation, the pots were randomly repositioned every three days.

### 4.3. Sampling and Analysis

#### 4.3.1. Plant Sampling

Plants were harvested at full maturity in both years. For the field experiment, 12 representative hills were collected from each plot. Each field plot was treated as one biological replicate and statistical unit. For the pot experiment, six pots per treatment were destructively sampled. Samples from two pots, comprising six plants in total, were composited to form one biological replicate, resulting in three biological replicates per treatment. Thus, for plant chemical analyses in the pot experiment, each two-pot composite represented one biological replicate and statistical unit. The sampled plants were rinsed with tap water and then washed three times with distilled water, followed by ultrapure water. Each plant was separated into the following parts: roots (Root), fourth (Fo), third (T), second (S), and first (Fi) basal stem sections, each comprising the node, internode, leaf, and sheath, and panicle, which was cut at the neck. The number of effective tillers per hill was recorded. Panicles were manually threshed to separate the rachis (Ra) from the grain. Filled and unfilled kernels were separated using the flotation method. All samples were first oven-dried at 110 °C for 30 min to deactivate enzymes and then dried at 70 °C until constant weight. Brown rice (BR) and husks (H) were obtained using a laboratory-scale husker. Field yield was determined by harvesting 100 hills from the central area of each plot. The harvested grains were threshed, sun-dried, and weighed, and the yield was adjusted to a standard moisture content of 13.5%.

#### 4.3.2. Cd Determination in Plant Tissues

The dried samples were ground into a fine powder and passed through a sieve. A representative subsample (0.2500 ± 0.0005 g) was digested on a graphite heating block using a mixture of concentrated HNO_3_ and HClO_4_ (9:1, *v*/*v*). Cd concentrations in the resulting digestate were determined by inductively coupled plasma mass spectrometry (ICP-MS; Agilent 7900, Agilent Technologies, Santa Clara, CA, USA). Quality assurance and quality control procedures were applied, including the analysis of reagent blanks, duplicate samples (one per 20 samples), and certified reference material (GBW07603-GSV-2) [39]. Cd recoveries ranged from 95% to 105%. Any sample with a deviation greater than 20% between duplicate measurements was re-prepared and reanalyzed. Additional methodological details are provided in Supplementary Test S1.

#### 4.3.3. Iron Plaque Extraction and Cd Adsorption

Iron plaques on root surfaces were extracted using the dithionite–citrate–bicarbonate (DCB) method, and the detailed protocol is provided in Supplementary Test S2. Concentrations of Cd and Fe in the DCB extracts were quantified simultaneously by ICP-MS. The amounts of Cd and Fe associated with iron plaques were calculated and expressed on a root dry-weight basis (mg kg^–1^) using the following formula: (concentration in the extract × extract volume)/root dry weight. After DCB extraction, the roots were dried, weighed, and stored for subsequent determination of internal Cd concentrations in root tissue.

#### 4.3.4. Soil Sampling and Analysis

At maturity, rhizosphere soil was collected by gently removing the soil tightly adhering to rice roots. A subsample of approximately 100 g fresh weight was immediately stored at −20 °C for subsequent determination of ammonium nitrogen (NH_4_^+^–N) and nitrate nitrogen (NO_3_^–^–N). The remaining soil was air-dried, ground, and passed through a sieve for analysis of other parameters. Soil pH was measured in a 1:2.5 (*w*/*v*) soil-water suspension. Available Cd (ACd) was extracted using 0.1 M CaCl_2_. Available phosphorus (AP) was determined using the molybdenum blue method. Exchangeable cations were extracted as follows: exchangeable calcium (E_Ca^2+^) and potassium (E_K^+^) were extracted with ammonium acetate, and exchangeable hydrogen (E_H^+^) was extracted with 1 M KCl and quantified by titration with NaOH. For mineral N analysis, NH_4_^+^–N and NO_3_^–^–N were extracted from the fresh soil subsample with 0.5 M KCl, and their concentrations were determined using a continuous-flow analyzer.

### 4.4. Data Processing and Statistical Analysis

Statistical analyses were performed using SPSS software (version 24.0; IBM Corp., Armonk, NY, USA). Two-way analysis of variance (ANOVA) was used to evaluate the effects of the experimental factors and their interactions. When significant effects were detected, means were compared using Tukey’s honestly significant difference (HSD) test at *p* < 0.05. To identify key factors affecting Cd accumulation in brown rice, random forest (RF) analysis was conducted using R software (version 4.3.3). Relationships between soil environmental factors and key plant Cd factors identified by RF analysis were further examined using Mantel tests implemented with the linkET package in R. Detailed parameters for the RF models and Mantel tests are provided in Supplementary Test S3.

The following indices were calculated to evaluate Cd accumulation and translocation. The Cd/Fe ratio in iron plaque was calculated as the ratio of DCB-extractable Cd to DCB-extractable Fe, with both expressed on a root dry-weight basis, as described in Section 4.3.3. The ratio was multiplied by 10,000 for statistical analysis and data presentation. The translocation factor (TF) between adjacent organs was calculated as: (TF_A–B) = [Cd]B/[Cd]A, where A and B represent source and sink organs, respectively. Cd accumulation in an organ = was calculated as Cd concentration × organ biomass. The flux-based root-to-shoot translocation factor (TF_Root–AP) was calculated as total Cd accumulation in aboveground organs, including husks and brown rice/total Cd accumulation in roots. Cd proportion in an organ was calculated as Cd accumulation in that organ/total Cd accumulation in the whole plant.

## 5. Conclusions

This study provides strong evidence that nitrogen fertilizer formulations play an important role in regulating Cd accumulation and yield development in rice. The main mechanism appears to involve two linked processes: nitrogen fertilizer formulations affect Cd bioavailability by changing rhizosphere pH and available Cd concentration, while also influencing Cd allocation by altering transport efficiency at important stem internodes and during redistribution from the panicle to the grain.

A clear and stable trade-off was observed. Nitrate-N consistently reduced Cd concentrations in brown rice (BR), but this reduction was accompanied by decreased yield. By contrast, ammonium-N maintained or even increased yield while substantially increasing Cd accumulation, whereas urea produced an intermediate response. Based on this mechanistic understanding, the combined pot and field experiments showed that improving nitrogen fertilizer management, such as applying urea or using nitrate–ammonium mixtures, can support a better balance between yield and Cd concentration in rice grains.

Collectively, these findings provide new insight into the physiological mechanisms underlying nitrogen formulation-mediated Cd accumulation and suggest practical and actionable nitrogen management strategies for achieving safe rice production in Cd-contaminated paddy systems.

Importantly, because nitrate-N and ammonium-N were supplied as calcium nitrate and ammonium sulfate, respectively, the observed effects may partly reflect the influence of accompanying Ca^2+^ and SO_4_^2–^ ions. Therefore, these findings should be interpreted with caution as the integrated outcomes of agronomically relevant nitrogen fertilizer formulations rather than the effects of nitrogen form alone.

## Data Availability

The original contributions presented in this study are included in the article. Further inquiries can be directed to the corresponding author.

## Supplementary Materials

The following supporting information can be downloaded at: https://www.mdpi.com/article/10.3390/plants15172618/s1, Test S1: Determination of Cd concentration in plant tissues; Test S2: Extraction of root iron plaque and determination of plaque-associated Cd and Fe concentrations; Test S3: Random forest analysis and Mantel test; Table S1: Effects of different nitrogen fertilizer formulations on Cd concentrations in various rice organs under pot conditions; Table S2: Effects of different nitrogen fertilizer formulations on Cd concentrations in various rice organs under field conditions; Table S3: F values from multifactorial ANOVA showing the effects of nitrogen fertilizer formulation, cultivar, year, cultivation environment, and their interactions on Cd concentrations in different rice organs; Table S4: Effects of different nitrogen fertilizer formulations on Cd translocation factors of rice under pot conditions; Table S5: Effects of different nitrogen fertilizer formulations on Cd translocation factors in rice under field conditions; Table S6: F values from multifactorial ANOVA showing the effects of nitrogen fertilizer formulation, cultivar, year, cultivation environment, and their interactions on Cd translocation factors in rice; Table S7: Effects of different nitrogen fertilizer formulations on Cd accumulation in various rice organs under pot conditions; Table S8: Effects of different nitrogen fertilizer formulations on Cd accumulation in various rice organs under field conditions; Table S9: F values from multifactorial ANOVA showing the effects of nitrogen fertilizer formulation, cultivar, year, cultivation environment, and their interactions on Cd accumulation in different rice organs; Table S10: Effects of different nitrogen fertilizer formulations on the proportion of Cd in various rice organs under pot conditions; Table S11: Effects of different nitrogen fertilizer formulations on the proportion of Cd in various rice organs under field conditions; Table S12: F values from multifactorial ANOVA showing the effects of nitrogen fertilizer formulation, cultivar, year, cultivation environment, and their interactions on proportion of Cd in various rice organs; Figure S1: Effects of nitrogen fertilizer formulations on soil available Cd content, pH, nitrate nitrogen, ammonium nitrogen, available phosphorus, exchangeable H^+^, exchangeable K^+^, and exchangeable Ca^2+^ under pot conditions.
