# Supplementary material for: Nitrogen Fertilizer Formulations Modulate the Yield–Cadmium Trade-Off in Rice Through Rhizosphere Processes and Translocation Nodes
Source: Plants (Basel). 2026 Aug 27;15(17):2618. doi: 10.3390/plants15172618 (PMC13567301; doi:10.3390/plants15172618)
Supplement: Supplementary file 1 [file plants-15-02618-s001.zip › plants-4382022-supplementary.pdf]

Supplementary information

Nitrogen Fertilizer Formulations Modulates the Yield–Cadmium Trade-Off in Rice Through Rhizosphere Processes and Translocation Nodes

Test: 3

Table: 12

Figure: 1

Test S1: Determination of Cd concentration in plant tissues

A subsample (0.2500 g ±0.0005 g) of dried and finely ground plant material was accurately weighed using an analytical balance and transferred into a 50 mL conical flask. Ten milliliters of a mixed concentrated acid solution (HNO_3:$HClO_4$ = 9:1, v/v; guaranteed reagent grade) was added. The mixture was allowed to predigest at room temperature in a fume hood for more than 10 hours.

The samples were subsequently digested in a programmable graphite-heating block. The temperature was first raised to 80 ℃ and maintained for 1 h, then gradually increased to 150–180 ℃ until the digest became clear or faintly yellow, and finally raised to 220 ℃ to evaporate the solution to approximately 1 mL.

After cooling to room temperature, the digest was transferred into a 50 mL volumetric flask and diluted to the volume with ultrapure water. The solution was shaken thoroughly, allowed to stand for approximately 30 min, and then filtered through a 0.45 µm syringe filter into centrifuge tubes. The concentrations of Cd and other mineral elements were determined using ICP-MS. Samples that were not analyzed immediately were stored at 4 ℃ before analysis.

The quality control procedures included one analytical batch comprising 80 samples, with four randomly selected samples analyzed in duplicate. Each batch also contained three procedural blanks and three certified reference materials (GBW07603-GSV-2, plants). The recoveries ranged from 95% to 105%. If the relative deviation between duplicate samples exceeded 20%, the entire batch was re-digested and reanalyzed. All glassware was soaked in 5% (v/v) HNO_3 for 24 h, rinsed with distilled and ultrapure water, and air-dried before use.

Test S2: Extraction of root iron plaque and determination of plaque-associated Cd and Fe concentrations

Iron plaques on the root surfaces were extracted using the dithionite–citrate–bicarbonate (DCB) method. Approximately 0.2000 g of intact, dried roots was weighed and placed in a 50 mL conical flask. Twenty milliliters of 0.3 M trisodium citrate ($Na_3C_6H_5O_7·2H_2O$), 2.5 mL of $NaHCO_3$ solution (0.1 M), and 0.5 g of sodium dithionite ($Na_2S_2O_4$) were added. The mixture was shaken at 25 °C for 3 h.

After extraction, the supernatant was transferred to a 100 mL volumetric flask for analysis. The roots were thoroughly rinsed with ultrapure water, and the rinsate was combined with the root extract. The solution was diluted to the desired volume with ultrapure water, shaken well, and allowed to stand for 30 min. The extract was then filtered, acidified with 5% (v/v) HNO_3 to stabilize Cd, and analyzed for Cd and Fe concentrations using ICP-MS.

Test S3: Random Forest analysis and Mantel test

Random Forest (RF) analysis

Random Forest (RF) analysis was employed to identify the key plant Cd-related factors associated with Cd accumulation in brown rice under pot and field cultivation conditions. For each cultivation system, the Cd concentrations in all measured plant organs (including iron plaque) and all calculated Cd translocation factors between adjacent organs were included as predictor variables. In contrast, the Cd concentration in brown rice was used as the response variable.

To improve the statistical robustness of the RF model and reduce the influence of year- and cultivar-specific variability, data from both experimental years (2022 and 2023) and both rice cultivars were pooled within each cultivation condition before model construction. All predictor variables were standardized (z-score transformation) before the analysis to ensure comparability among variables with different units and magnitudes.

The RF analysis was conducted using the Random Forest package in R (version 4.3.3). The number of trees (ntree) was set to 1000, which was sufficient to stabilize the out-of-bag (OOB) error. The number of variables randomly sampled at each split (mtry)

was optimized using the default setting (i.e., the square root of the total number of predictors) and verified by minimizing the OOB error. The node size was set to the default value for the regression models.

Model performance was evaluated using OOB error estimates, and variable importance was quantified based on the percentage increase in the mean squared error (%IncMSE). Variables with higher %IncMSE values were considered to have stronger associations with the Cd concentration in brown rice. The top-ranked variables were defined as the most influential factors and were subsequently used for further evaluations.

It should be emphasized that the RF analysis in this study was applied for variable screening and importance ranking, rather than for direct causal inference or predictive modeling.

Mantel test

To explore potential associations between soil environmental factors and key plant Cd accumulation factors identified by RF analysis, Mantel tests were performed using the linkET package in R.

Prior to analysis, soil and plant variables were log- or z-score transformed as appropriate to reduce scale effects and improve normality. Distance matrices were constructed using the Euclidean distance for both soil environmental factors and plant Cd-related variables. Mantel tests were conducted with 999 permutations to assess the statistical significance of the correlations between the distance matrices.

The Mantel correlation coefficient (r) was used to quantify the strength of the association, and significance was determined at $P < 0.05$. The Mantel test was applied to the combined datasets (pooled across years and cultivars) under pot conditions, consistent with the RF analysis, to focus on the overall relationships between rhizosphere soil properties and plant Cd accumulation processes.

Table S1 Effects of different nitrogen fertilizer formulations on Cd concentrations in various rice organs under pot conditions.

Year	Cultivar	Treatment	RP	Root	Fo	T	S	Fi	Ra	H	BR
2022	X13	U	5.18a	0.31b	1.24a	1.49a	1.32a	1.76a	0.88a	0.28a	0.056b
		N	1.85b	0.23b	0.41c	0.18c	0.14c	0.17c	0.07c	0.03c	0.037c
		A	5.35a	0.45a	0.94b	0.88b	0.62b	0.81b	0.42b	0.10b	0.066a
	YZX	U	2.95c	0.88a	0.71c	0.38b	0.24b	0.22b	0.19b	0.07b	0.087b
		N	4.46b	0.8a	0.81b	0.39b	0.27b	0.24b	0.16b	0.08b	0.065c
		A	6.13a	1.00a	0.97a	0.92a	2.61a	1.81a	0.72a	0.25a	0.138a
2023	X13	U	1.35b	1.41b	0.16b	0.07a	0.09b	0.08b	0.10b	0.05b	0.031b
		N	0.57b	0.28c	0.10c	0.08a	0.10b	0.05c	0.28a	0.03b	0.009c
		A	7.77a	1.74a	0.77a	0.50a	0.53a	0.42a	0.09b	0.15a	0.05a
	YZX	U	5.54ab	2.15a	0.20b	0.15b	0.14b	0.18b	0.23b	0.14b	0.046b
		N	4.01b	1.84a	0.12c	0.09c	0.04c	0.20b	0.80a	0.11b	0.028c
		A	6.10a	1.54a	0.78a	0.77a	0.57a	0.73a	0.14c	0.32a	0.071a

Values are means (n=3). Different lowercase letters indicate significant differences among nitrogen form treatments within the same cultivar at $p < 0.05$.

RP, iron plaque; R, root; Fo, fourth basal stem section; T, third basal stem section; S, second basal stem section; Fi, first basal stem section; Ra, rachis; H, husk; BR, brown rice; U, urea; N, nitrate-N; A, ammonium-N. X13, Xiangwanxian 13 hao; YZX, Yuzhenxiang.

Table S2 Effects of different nitrogen fertilizer formulations on Cd concentrations in various rice organs under field conditions

Year	Cultivar	Treatment	RP	Root	Fo	T	S	Fi	Ra	H	BR
2022	X13	U	3.97b	0.84b	0.78b	0.72b	0.80c	0.81c	0.40a	0.09a	0.108b
		N	4.82a	1.52a	0.86a	0.16c	0.93b	1.32a	0.34a	0.10a	0.096b
		A	3.72b	1.17ab	0.54c	0.95a	1.01a	1.13b	0.33a	0.09a	0.164a
	YZX	U	1.18b	0.32b	0.52b	0.53c	0.38c	0.07c	0.38b	0.02c	0.145b
		N	1.25b	0.15c	0.24c	1.07a	0.64b	0.21b	0.17c	0.05b	0.015c
		A	2.79a	0.58a	0.75a	0.67b	0.86a	0.91a	0.46a	0.10a	0.181a
2023	X13	U	6.26b	1.24b	1.25b	1.00c	0.60c	1.02c	0.84c	0.60c	0.129b
		N	6.39b	1.33b	1.19b	1.35b	0.90a	1.57a	1.62a	1.27a	0.112b
		A	15.83a	2.47a	1.47a	1.65a	0.78b	1.15b	1.05b	0.70b	0.176a
	YZX	U	6.55c	1.05b	0.97c	1.09c	0.46c	0.48c	0.51c	0.37c	0.209b
		N	21.45a	4.13a	1.39a	1.42a	0.63a	1.33a	2.10a	0.57a	0.20b
		A	10.03b	1.49b	1.12b	1.18b	0.53b	0.52b	1.31b	0.45b	0.297a

Values are means (n=3). Different lowercase letters indicate significant differences among nitrogen form treatments within the same cultivar at $p < 0.05$.

RP, iron plaque; R, root; Fo, fourth basal stem section; T, third basal stem section; S, second basal stem section; Fi, first basal stem section; Ra, rachis; H, husk; BR, brown rice; U, urea; N, nitrate-N; A, ammonium-N. X13, Xiangwanxian 13 hao; YZX, Yuzhenxiang.

Table S3 F values from multifactorial ANOVA showing the effects of nitrogen fertilizer formulations (N), cultivar (C), year (Y), cultivation environment (E), and their interaction (N × E) on Cd concentrations in different rice organs.

Source of variation	RP	Root	Fo	T	S	Fi	Ra	H	BR
Nitrogen fertilizer formulations (N)	58.48***	6.69**	25.49***	26.06***	72.33***	23.3***	37.76***	35.33***	123.88***
Cultivar	12.76**	13.45**	4.65*	ns	ns	24.31***	9.97**	72.08***	98.7***
Year	285.98***	289.08***	6.47*	7.28*	83.24***	4.41*	236.42***	1096.57***	21.25***
Environment (E)	136.7***	21.89***	104.2***	144.76***	22.77***	49.45***	331.66***	697.73***	639.83***
N × E	37.55***	45.39***	15.86***	11.98***	50.74***	34.31***	31.48***	117.02***	16.06***

*, **, *** indicate significance at $p < 0.05$, 0.01 and 0.001, respectively; ns, not significant. RP, iron plaque; R, root; Fo, fourth basal stem section; T, third basal stem section; S, second basal stem section; Fi, first basal stem section; Ra, rachis; H, husk; BR, brown rice.

Table S4 Effects of different nitrogen fertilizer formulations on Cd translocation factors of rice under pot conditions

Year	Cultivar	Treatment	TF_RP-root	TF_Root-Fo	TF_Fo-T	TF_T-S	TF_S-Fi	TF_Fi-Ra	TF_Ra-H	TF_H-BR	TF_Ra-BR	TF_Root-AP
2022	X13	U	0.06b	3.99a	1.20a	0.88a	1.34a	0.50ab	0.32b	0.20c	0.06c	29.95a
		N	0.12a	1.88b	0.43b	0.78ab	1.22a	0.39b	0.47a	1.22a	0.57a	9.86c
		A	0.09ab	2.24b	0.93c	0.70b	1.31a	0.53a	0.24b	0.66b	0.16b	19.94b
	YZX	U	0.31a	0.80a	0.53b	0.63b	0.91a	0.88a	0.35b	1.33a	0.46a	5.05b
		N	0.18b	1.04a	0.48c	0.68b	0.91a	0.69a	0.48a	0.82b	0.40a	4.69b
		A	0.16b	0.98a	0.95a	2.84a	0.69b	0.40b	0.34b	0.56b	0.19b	20.01a
2023	X13	U	1.06a	0.11b	0.45b	1.21a	1.00a	1.17b	0.51b	0.63a	0.32b	0.62c
		N	0.49b	0.37a	0.86a	1.23a	0.53c	5.34a	0.12c	0.28b	0.03c	1.43b
		A	0.23c	0.44a	0.66ab	1.05a	0.79b	0.22b	1.67a	0.34b	0.56a	3.49a
	YZX	U	0.39a	0.09b	0.77b	0.91a	1.30b	1.31b	0.58b	0.35a	0.20b	0.46b
		N	0.45a	0.07b	0.75b	0.46c	4.91a	4.08a	0.14c	0.25ab	0.04c	0.35b
		A	0.26b	0.52a	0.99a	0.74b	1.27b	0.20c	2.31a	0.22b	0.51a	2.70a

Values are means (n=3). Different lowercase letters indicate significant differences among nitrogen form treatments within the same cultivar at $p < 0.05$.

RP, iron plaque; R, root; Fo, fourth basal stem section; T, third basal stem section; S, second basal stem section; Fi, first basal stem section; Ra, rachis; H, husk; BR, brown rice; TF, translocation factor; U, urea; N, nitrate-N; A, ammonium-N. X13, Xiangwanxian 13 hao; YZX, Yuzhenxiang. TF, transfer factor; TF_Root-AP is the flux-based root-to-aboveground parts translocation factor, calculated as the ratio of total Cd accumulation in aboveground parts (including husks, and brown rice) to that in roots. Cd accumulation in each organ was obtained by multiplying Cd concentration by organ biomass.

Table S5 Effects of different nitrogen fertilizer formulations on Cd translocation factors in rice under field conditions

Year	Cultivar	Treatment	TF_RP-root	TF_Root-Fo	TF_Fo-T	TF_T-S	TF_S-Fi	TF_Fi-Ra	TF_Ra-H	TF_H-BR	TF_Ra-BR	TF_Root-AP
2022	X13	U	0.21a	0.93a	0.93b	1.12b	1.02b	0.49a	0.23a	1.20b	0.28b	20.69a
		N	0.31a	0.6b	0.19c	5.67a	1.42a	0.26b	0.29a	0.98b	0.28b	13.87b
		A	0.31a	0.48b	1.76a	1.07b	1.12b	0.29b	0.27a	1.87a	0.50a	15.00b
	YZX	U	0.27a	1.62a	1.03b	0.72b	0.18c	5.88a	0.05c	8.62a	0.38a	21.92c
		N	0.12b	1.62a	4.52a	0.60b	0.33b	0.83b	0.30a	0.29b	0.08b	59.60a
		A	0.21a	1.30a	0.90b	1.28a	1.06a	0.51c	0.21b	1.94b	0.39a	35.85b
2023	X13	U	0.20a	1.02a	0.80a	0.60b	1.72a	0.82b	0.72ab	0.21a	0.15a	20.01a
		N	0.21a	0.93a	1.13b	0.66a	1.75a	1.03a	0.78a	0.09b	0.07b	23.39a
		A	0.16b	0.61b	1.12a	0.47c	1.48b	0.91b	0.67b	0.25a	0.17a	8.41b
	YZX	U	0.17a	0.93a	1.12a	0.42a	1.06b	1.06c	0.73a	0.57a	0.41a	17.18a
		N	0.19a	0.34b	1.02b	0.45a	2.10a	1.58b	0.27b	0.36b	0.10c	6.78b
		A	0.15a	0.77a	1.06ab	0.45a	0.98b	2.52a	0.35b	0.66a	0.23b	9.20b

Values are means (n=3). Different lowercase letters indicate significant differences among nitrogen form treatments within the same cultivar at $p < 0.05$.

RP, iron plaque; R, root; Fo, fourth basal stem section; T, third basal stem section; S, second basal stem section; Fi, first basal stem section; Ra, rachis; H, husk; BR, brown rice; TF, translocation factor; U, urea; N, nitrate-N; A, ammonium-N. X13, Xiangwanxian 13 hao; YZX, Yuzhenxiang. TF, transfer factor; TF_Root-AP is the flux-based root-to-aboveground parts (including husks, and brown rice) translocation factor, calculated as the ratio of total Cd accumulation in aboveground parts (including husks, and brown rice) to that in roots. Cd accumulation in each organ was obtained by multiplying Cd concentration by organ biomass.

Table S6 F values from multifactorial ANOVA showing the effects of nitrogen fertilizer formulations (N), cultivar (C), year (Y), cultivation environment (E), and their interaction (N × E) on Cd translocation factors in rice

Source of variation	TF_RP-root	TF_Root-Fo	TF_Fo-T	TF_T-S	TF_S-Fi	TF_Fi-Ra	TF_Ra-H	TF_H-BR	TF_Ra-BR	TF_Root-AP
Nitrogen fertilizer formulations (N)	34.39***	12.99***	57.90***	197.33***	26.45***	64.78***	110.95***	29.31***	85.00***	0.43ns
Cultivar	12.55**	26.83***	158.43***	445.76***	ns	67.84***	0.49ns	29.84***	4.31*	5.34*
Year	93.24***	275.58***	115.40***	1120.94***	69.62***	78.60***	363.02***	110.97***	83.6***	480.07***
Environment (E)	62.03***	4.22*	513.71***	31.31***	4.99*	ns	93.38***	47.88***	18.33***	427.90***
N × E	32.28***	2.15ns	124.34***	536.39***	6.19**	117.99***	139.18***	28.19***	29.97***	59.86***

*, **, *** indicate significance at $p < 0.05$, 0.01 and 0.001, respectively; ns, not significant. RP, iron plaque; R, root; Fo, fourth basal stem section; T, third basal stem section; S, second basal stem section; Fi, first basal stem section; Ra, rachis; H, husk; BR, brown rice; TF, translocation factor; U, urea; N, nitrate-N; A, ammonium-N. X13, Xiangwanxian 13 hao; YZX, Yuzhenxiang. TF, transfer factor; TF_Root-AP is the flux-based root-to-shoot translocation factor, calculated as the ratio of total Cd accumulation in aboveground parts (including husks, and brown rice) to that in roots. Cd accumulation in each organ was obtained by multiplying Cd concentration by organ biomass.

Table S7 Effects of different nitrogen fertilizer formulations on Cd accumulation in various rice organs under pot conditions.

Year	Cultivar	Treatment	RP	Root	Fo	T	S	Fi	Ra	H	BR
2022	X13	U	27.75a	1.66a	10.88a	10.53a	9.43a	13.11a	1.93a	1.85a	1.68b
		N	6.33c	0.78b	2.68b	0.93c	0.73c	0.96c	0.12c	0.17c	0.89c
		A	16.12b	1.38ab	10.3a	7.31b	5.09b	6.8b	1.11b	0.74b	2.3a
	YZX	U	12.64b	3.76a	9.07b	3.42b	2.31b	1.73b	0.41b	0.35b	1.71ab
		N	16.35b	2.94a	7.77b	2.99b	1.89b	1.52b	0.29b	0.38b	1.15b
		A	24.34a	3.98a	14.78a	10.32a	29.01a	18.24a	1.72a	1.38a	2.69a
2023	X13	U	8.7b	9.01a	2.21b	0.69b	0.8b	0.67b	0.21c	0.29b	0.64b
		N	4.62c	2.24b	1.08c	0.63b	0.79b	0.38b	0.53a	0.11b	0.11c
		A	42.52a	9.63a	15.08a	7.81a	8.51a	5.67a	0.29b	0.85a	1.04a
	YZX	U	58.33a	22.32a	3.22b	1.77b	1.49b	1.49b	0.45b	0.76b	0.96b
		N	29.49b	13.67b	1.32b	0.65b	0.29b	1.22b	1.21a	0.64b	0.61c
		A	60.8a	15.34b	17.6a	13.38a	9.46a	10.6a	0.41b	1.65a	1.43a

Values are means (n=3). Different lowercase letters indicate significant differences among nitrogen form treatments within the same cultivar at $p < 0.05$. RP, iron plaque; R, root; Fo, fourth basal stem section; T, third basal stem section; S, second basal stem section; Fi, first basal stem section; Ra, rachis; H, husk; BR, brown rice; U, urea; N, nitrate-N; A, ammonium-N. X13, Xiangwanxian 13 hao; YZX, Yuzhenxiang.

Table S8 Effects of different nitrogen fertilizer formulations on Cd accumulation in various rice organs under field conditions.

Year	Cultivar	Treatment	RP	Root	Fo	T	S	Fi	Ra	H	BR
2022	X13	U	194.75a	41.04b	234.67a	157.79a	175.09b	215.08a	12.57a	11.27a	40.62b
		N	200.87a	64.83a	271.63a	27.17b	151.78b	218.96a	7.9b	8.43a	27.89c
		A	211.06a	64.66a	183.01b	181.96a	286.93a	222.05a	9.76ab	9.55a	59.95a
	YZX	U	70.99b	18.95b	116.23b	111.54b	81.75b	13.98c	16.29b	3.04c	70.92b
		N	57.33b	7.04c	44.37c	191.38a	119.25b	38.52b	7.11c	7.57b	5.33c
		A	135.33a	27.76a	180.14a	145.4b	208.25a	202.58a	24.11a	17.29a	96.86a
2023	X13	U	175.95b	34.67b	229.41b	124.16c	75.18b	128.03b	23.91c	59.63b	49.37b
		N	232.36b	48.33b	170.14c	187.68b	117.95a	225.6a	57.21a	135.3a	45.19b
		A	844.1a	131.76a	295.97a	260.28a	127.62a	192.66a	43.17b	77.07b	80.66a
	YZX	U	212.31b	33.23b	174.23a	120.99b	58.31b	62.02b	16.34c	43.58b	89.57b
		N	624.99a	120.62a	208.01a	140.92ab	71b	183.05a	58.13a	65.63a	81.89b
		A	656.66a	97.92a	224.94a	170.53a	86.17a	74.92b	43.05b	60.26a	142.95a

Values are means (n=3). Different lowercase letters indicate significant differences among nitrogen form treatments within the same cultivar at $p < 0.05$. RP, iron plaque; R, root; Fo, fourth basal stem section; T, third basal stem section; S, second basal stem section; Fi, first basal stem section; Ra, rachis; H, husk; BR, brown rice; U, urea; N, nitrate-N; A, ammonium-N. X13, Xiangwanxian 13 hao; YZX, Yuzhenxiang.

Table S9 F values from multifactorial ANOVA showing the effects of nitrogen fertilizer formulations (N), cultivar (C), year (Y), cultivation environment (E), and their interaction (N × E) on Cd accumulation in different rice organs.

Source of variation	RP	Root	Fo	T	S	Fi	Ra	H	BR
Nitrogen fertilizer formulations (N)	100.6***	30.89***	9.28***	35.63***	43.26***	28.33***	113.96***	49.58***	127.78***
Cultivar	0ns	2.11ns	38.76***	1.56ns	33.36***	147.98***	4.16ns	73.43***	115.07***
Year	334.54***	105.08***	14.22***	17.37***	104.27***	1.74ns	933.49***	1024.79***	111.02***
Environment (E)	937.07***	426.16***	1071.62***	1682.38***	875.71***	1131.9***	3455.09***	1667***	2008.63***
N × E	86.28***	36.29***	3.51ns	22.38***	24.75***	24.94***	116.84***	53.82***	117.41***

*, **, *** indicate significance at $p < 0.05$, 0.01 and 0.001, respectively; ns, not significant. RP, iron plaque; R, root; Fo, fourth basal stem section; T, third basal stem section; S, second basal stem section; Fi, first basal stem section; Ra, rachis; H, husk; BR, brown rice.

Table S10 Effects of different nitrogen fertilizer formulations on the proportion of Cd in various rice organs under pot conditions.

Year	Cultivar	Treatment	RP	Root	Fo	T	S	Fi	Ra	H	BR
2022	X13	U	34.76b	2.11b	13.99b	13.42a	12a	16.74a	2.45a	2.39a	2.15c
		N	46.52a	5.72a	19.78a	6.87b	5.38c	7.07c	0.88b	1.23b	6.56a
		A	31.48b	2.69b	20.18a	14.29a	9.96b	13.3b	2.17a	1.45b	4.5b
	YZX	U	35.37b	10.65a	25.62a	9.82a	6.55b	4.89b	1.16b	1.01a	4.93a
		N	46.37a	8.4a	21.88a	8.49a	5.37c	4.32b	0.81c	1.09a	3.27a
		A	22.85c	3.74b	13.87b	9.69a	27.27a	17.13a	1.61a	1.3a	2.53a
2023	X13	U	37.21a	38.7a	9.79b	2.96c	3.49b	2.94b	0.91b	1.23a	2.77a
		N	43.6a	21.17b	10.61ab	6.12b	7.67a	3.58b	5.13a	1.05ab	1.06b
		A	46.2a	10.59c	16.66a	8.6a	9.35a	6.21a	0.32b	0.93b	1.14b
	YZX	U	63.79a	24.81a	3.65b	1.95b	1.64b	1.66b	0.51b	0.89a	1.11a
		N	59.97a	27.19a	2.84b	1.4b	0.6b	2.71b	2.59a	1.4a	1.29a
		A	44.9b	11.5b	14.18a	10.41a	7.83a	8.31a	0.31b	1.36a	1.19a

Values are means (n=3). Different lowercase letters indicate significant differences among nitrogen form treatments within the same cultivar at $p < 0.05$. RP, iron plaque; R, root; Fo, fourth basal stem section; T, third basal stem section; S, second basal stem section; Fi, first basal stem section; Ra, rachis; H, husk; BR, brown rice; U, urea; N, nitrate-N; A, ammonium-N. X13, Xiangwanxian 13 hao; YZX, Yuzhenxiang.

Table S11 Effects of different nitrogen fertilizer formulations on the proportion of Cd in various rice organs under field conditions.

Year	Cultivar	Treatment	RP	Root	Fo	T	S	Fi	Ra	H	BR
2022	X13	U	17.93a	3.8b	21.78a	14.48a	16.16a	19.9a	1.16a	1.03a	3.74b
		N	20.48a	6.48a	27.76a	2.79b	15.58a	22.28a	0.81b	0.89a	2.93ab
		A	17.05a	5.28ab	14.7b	14.74a	23.82a	17.93a	0.79b	0.79a	4.91a
	YZX	U	14.06a	3.77a	23.08a	22.05b	16.02c	2.79c	3.26a	0.63b	14.34a
		N	12.02a	1.48c	9.27c	39.97c	24.99a	8.06b	1.49c	1.6a	1.12c
		A	13.09a	2.69b	17.27b	14.01a	19.98b	19.59a	2.32b	1.68a	9.37b
2023	X13	U	19.52b	3.86b	25.48a	13.77ab	8.32b	14.21b	2.68b	6.65b	5.51a
		N	19.07b	3.97b	13.96b	15.35a	9.65a	18.46a	4.7a	11.13a	3.71b
		A	41.05a	6.41a	14.42b	12.71b	6.22c	9.4c	2.11b	3.75c	3.93b
	YZX	U	25.83b	4.13b	21.38a	15.01a	7.21a	7.73b	2.03c	5.4a	11.28a
		N	40.19a	7.75a	13.28b	9.09b	4.59b	11.83a	3.74b	4.25b	5.27b
		A	42a	6.3a	14.48b	11.03b	5.58b	4.77c	2.78a	3.89b	9.16a

Values are means (n=3). Different lowercase letters indicate significant differences among nitrogen form treatments within the same cultivar at $p < 0.05$. RP, iron plaque; R, root; Fo, fourth basal stem section; T, third basal stem section; S, second basal stem section; Fi, first basal stem section; Ra, rachis; H, husk; BR, brown rice; U, urea; N, nitrate-N; A, ammonium-N. X13, Xiangwanxian 13 hao; YZX, Yuzhenxiang.

Table S12 F values from multifactorial ANOVA showing the effects of nitrogen fertilizer formulations (N), cultivar (C), year (Y), cultivation environment (E), and their interaction (N × E) on proportion of Cd in various rice organs.

Source of variation	RP	Root	Fo	T	S	Fi	Ra	H	BR
Nitrogen fertilizer formulations (N)	8.41**	78.12**	8.82**	1.44ns	44.02***	35.7***	50.75***	21.24***	31.41***
Cultivar	13.64**	0.14ns	13.5**	46.53***	0ns	229.06***	2.26ns	32.3***	47.08***
Year	192.81***	625.44***	79.06***	249.71***	385.86***	261.33***	81.2***	363.43***	16.36***
Environment (E)	349.52***	646.25***	32.36***	534.32***	116.76***	313.74***	83.23***	350.31***	179.63***
N × E	30.05***	112.99***	24.17***	65.51***	29.09***	69.34***	6.75**	24.82***	42.23***

*, **, *** indicate significance at $p < 0.05$, 0.01 and 0.001, respectively; ns, not significant. RP, iron plaque; R, root; Fo, fourth basal stem section; T, third basal stem section; S, second basal stem section; Fi, first basal stem section; Ra, rachis; H, husk; BR, brown rice.

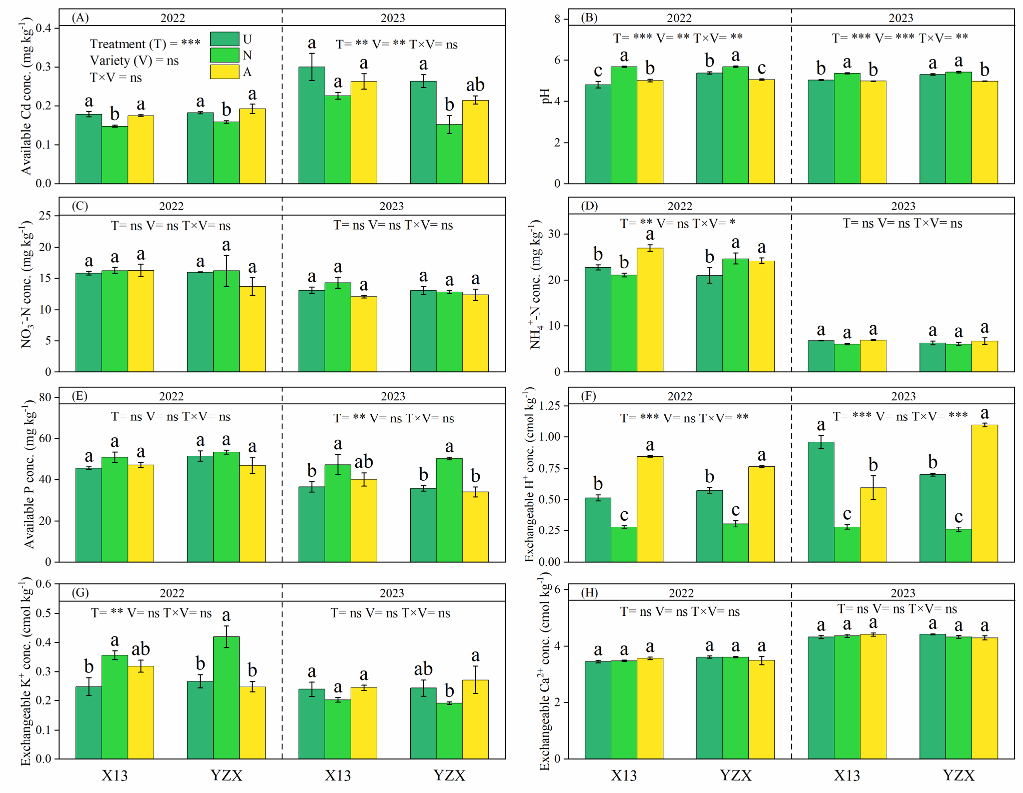

Figure S1 Effects of nitrogen fertilizer formulations on soil available Cd content (A), pH(B), nitrate nitrogen (C), ammonium nitrogen (D), available phosphorus (E), exchangeable H^+(F), exchangeable K^+(G), and exchangeable Ca^{2+}(H) under pot conditions. U, urea; N, nitrate-N; A, ammonium-N. X13, Xiangwanxian 13 hao; YZX, Yuzhenxiang. *,**,*** represent significance at 0.05, 0.01, and 0.001 levels, respectively; ns represents $p > 0.05$.
